# PVDF/PGMA Blend Membranes: NIPS-Driven Microstructure, Thermodynamic Miscibility, and Enhanced Wettability

**DOI:** 10.1021/acsomega.5c08866

**Published:** 2026-01-21

**Authors:** Md. Azizul Hakim, Md. Mahadi Hasan, Md. Al-Mamun, Md. Shamim Hossan, A. A. S. Mostofa Zahid, M. Habibur Rahman

**Affiliations:** † Department of Chemistry, 118869 University of Rajshahi, Rajshahi 6205, Bangladesh; ‡ Materials Science Division, Atomic Energy Center, Bangladesh Atomic Energy Commission, Dhaka 1207, Bangladesh; § Department of Nutrition and Food Engineering, Daffodil International University, Birulia, Dhaka 1216, Bangladesh

## Abstract

Novel poly­(vinylidene fluoride) (PVDF)/poly­(glycidyl methacrylate) (PGMA) blend membranes were successfully fabricated via the nonsolvent-induced phase separation (NIPS) process. Theoretical prediction using the Schneier equation and comprehensive characterization (XRD, FTIR, DSC, FESEM) established that the system exhibits partial miscibility with a critical phase separation threshold around 37 vol % PGMA. Below this threshold, good polymer miscibility was evidenced by favorable thermodynamic parameters and spectroscopic shifts. Beyond it, clear macroscopic phase separation occurred, influencing crystal uniformity and morphology. Crucially, the NIPS process strongly promoted the crystallization of PVDF into the polar, electroactive β-phase. The β-phase content was significantly enhanced from 17% in neat PVDF powder to a maximum of 70% in the optimized blend, despite the overall degree of crystallinity remaining relatively low (26–31%). Moreover, melting the NIPS-formed blends further enhanced the β-phase content at low-to-mid PGMA concentrations. Beyond the crystalline phase modulation, PGMA incorporation effectively modulated the membrane microstructure, significantly enhancing both porosity and surface hydrophilicity. The demonstrated ability to tune the microstructure and polar phase formation through simple blend composition makes these PVDF/PGMA membranes highly promising candidates for advanced functional and biomedical applications.

## Graphical abstract

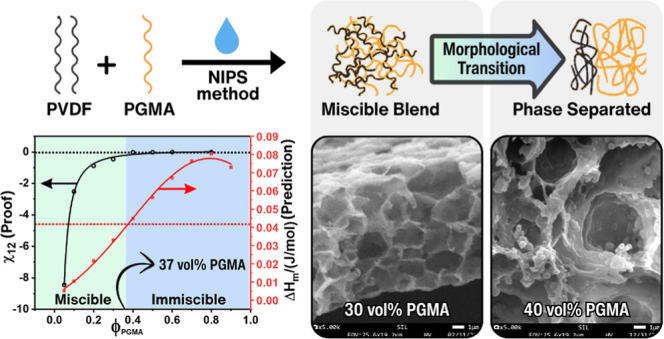

## 1 Introduction

Polymers have become indispensable engineering materials, surpassing traditional metals and ceramics due to their inherent flexibility, exceptional corrosion resistance, low cost, and superior specific strength—vital for aerospace, automotive, and advanced biomedical sectors. While the unique physical and chemical traits of individual polymers are well-established, the conventional approach of synthesizing entirely new polymers for every specific application is often an inefficient path, being both complex and prohibitively time-consuming.^1^ Consequently, materials science has shifted focus to combinatorial strategies that modify or merge existing macromolecules to achieve tailored, synergistic performance. These strategies, which include block/graft copolymerization,^2,3^ interpenetrating polymer network (IPN) formation,^4^ and polymer blending^5^ have proven highly effective. Among these methods, polymer blending remains exceptionally attractive due to its simplicity, efficiency, and versatility in rapidly creating new materials with precisely engineered properties, bypassing the need for novel monomer or polymer synthesis.^6,7^

A polymer blend is a physical mixture of two or more polymers designed to combine their advantageous properties into a single material.^8^ By selecting appropriate components, one can tailor properties such as toughness, elasticity,^9^ thermal stability,^10^ electrical conductivity,^11^ and hydrophilicity for diverse applications, including membranes, sensors,^12–14^ and biomedical devices.^15–18^ The properties of blends can be tailored by carefully choosing the component polymers.^19^ The major challenge, however, lies in achieving thermodynamic compatibility between dissimilar polymers. Differences in chain polarity, molecular structure, and lack of favorable intermolecular interactions frequently result in phase separation, which can deteriorate material performance. In practice, most polymers are either immiscible or partially miscible because the entropy of mixing is small and rarely compensates for unfavorable enthalpic interactions between unlike chains.^8,20–22^

Polymer miscibility can be evaluated using several indicators, including the presence of a single glass transition temperature (*T*_g_), melting point depression, a negative Flory–Huggins interaction parameter (χ_12_), homogeneous phase structure, and negative Gibbs free energy of mixing.^23^ Complete miscibility is uncommon; notable examples include PVPh/PVA,^24^ PVPh/PVMK,^25^ PVME/PS,^26^ PEO/PMMA,^27^ PLLA/PVPh,^28^ PVDF/PMMA,^29^ TPU/PC,^30^ PVDF/PBA,^31^ PMMA/PS,^32^ and PET/PVDF^33^ systems. Nevertheless, many systems exhibit partial miscibility, where compatibility is sensitively dependent on composition and temperature.

Polymer blends are broadly classified based on the phase state of their components as amorphous/amorphous, amorphous/crystalline, or crystalline/crystalline.^6^ Among these, crystalline–amorphous blends are particularly fascinating because the intimate microstructural interaction between ordered and disordered phases provides a powerful route to simultaneously tailor mechanical, dielectric, and barrier properties.

Poly­(vinylidene fluoride) (PVDF) is a semicrystalline polymer^34^ celebrated for its exceptional thermal stability, chemical resistance,^35^ mechanical strength,^36^ and electroactive properties, including piezoelectricity, pyroelectricity, and ferroelectricity.^37,38^ These desirable properties originate from PVDF’s polymorphism, as the polymer can crystallize into five distinct forms: α, β, γ, δ, and ε.^29^ The nonpolar α-phase (TGTG′) is the most stable but lacks electroactivity. Conversely, the highly polar β-phase (all-trans conformation) exhibits the strongest electroactive behavior, while the γ-phase (TTTGTTTG′) shows intermediate polarity.^39–41^ Achieving high β-phase content is crucial for electroactive and sensing applications. However, since PVDF preferentially crystallizes into the nonpolar α-phase, researchers rely on strategies such as adjusting fabrication parameters (e.g., temperature, solvent, and concentration),^42^ adding specific additives or utilizing advanced processing techniques.^43^ Notably, the nonsolvent-induced phase separation (NIPS) is especially effective for simultaneously producing porous membranes and achieving high β-phase content, making them highly suitable for industrial separation and biomedical applications.^44^

Despite its remarkable qualities, PVDF suffers from several notable limitations, primarily its inherent hydrophobicity (rendering it prone to fouling in filtration applications),^60^ limited surface reactive groups, susceptibility to radiative damage in harsh environments (such as space),^61^ and its nonbiodegradable nature.^62^ Blending PVDF with a second polymer is the most effective strategy to overcome these drawbacks and tailor surface functionality. However, due to its high degree of crystallinity and chemical inertness, achieving full compatibility when blending PVDF often presents significant miscibility challenges. Nonetheless, PVDF has demonstrated promising miscibility and partial miscibility with a select number of amorphous and crystalline polymers, a detailed overview of which is provided in Table 1. The mechanism driving miscibility in these PVDF-based blends generally relies on favorable dipole–dipole interactions and/or hydrogen bonding.^63^ Specifically, PVDF often exhibits miscibility with polar, amorphous, carbonyl-containing polymers—such as poly­(methyl methacrylate) (PMMA), poly­(ethyl methacrylate) (PEMA), poly­(vinyl methyl ketone) (PVMK), and poly­(methoxymethyl methacrylate) (PMOMA)—due to strong dipolar interactions between the highly electronegative fluorine atoms (−CF_2_−) on the PVDF chain and the polar carbonyl groups (>C=O) of the blending partner.^64^ A similar principle governs miscibility in PVDF/PAN blends, where the nitrile groups (C≡N) form strong dipole–dipole interaction with PVDF chains. Consequently, the presence of polar functional groups such as ester, ketone, ether, nitrile, amide, or sulfonic acid in the blending partner is crucial for achieving compatibility with PVDF.^45,65,66^ Despite this broad range of possibilities, PMMA remains the most prominently studied polymer, demonstrating complete miscibility with PVDF.

**Table 1:** List of Polymers Completely and Partially Miscible with PVDF

partner polymer	miscibility	processing technique	properties & miscibility criteria	ref
PEI	partial	NIPS	Enhanced hydrophilicity, porosity, mechanical strength, and pure water flux, alongside high oil rejection	^45^
PEO^a^	partial	solution mixing	Negative χ_12_ for PVDF-rich blends	^46,47^
PMMA	complete	melt-blending and extrusion calendaring	Improved energy storage properties with 50% PMMA content; single *T*_g_, negative χ_12_ through all compositions	^48,49^
PET^a^	complete	melt-blending	Single *T*_g_ and depressed melting point	^50,51^
PBA^a^	complete	melt-blending	Negative value of χ_12_ and single *T*_g_	^52,53^
PVP	complete	solution casting	High proton conductivity, excellent flowability; Negative χ_12_	^54–56^
PCL^a^	partial	solution casting	Miscible up to 30 wt % PVDF content; cloud point, and melting point depression	^6,57^
PVAc	complete	solution casting	Higher β-phase fraction, stronger piezoelectric effects; single *T*_g_ and depressed melting point	^21,22,58^
PMOMA	complete	solution casting	Single *T*_g_, melting point depression, and negative value of χ_12_	^59^
PGMA	partial	NIPS	Miscible up to ca. 37 vol % PGMA, better hydrophilicity and porosity; negative χ_12_	This work

a Crystalline/Crystalline Polymer Blend.

**Table 2:** Composition and Designations of the NIPS-Formed PVDF/PGMA Blend Membrane Samples

designation^a^	PVDF	0PG	5PG	10PG	20PG	30PG	40PG	50PG	60PG	80PG	100PG
vol % PGMA	—	0	5	10	20	30	40	50	60	80	100
vol % PVDF	—	100	95	90	80	70	60	50	40	20	0

a PVDF/PGMA blends are designated as xPG, where the number (*x*) represents the volume percentage (vol %) of PGMA in the blend. The as-received PVDF powder is designated simply as PVDF.

Among polar amorphous polymers, poly­(glycidyl methacrylate) (PGMA) is a promising candidate for blending with PVDF. It shares the same desirable methacrylate backbone as PMMA but further contains reactive epoxy side groups that enable subsequent chemical modification or cross-linking.^67,68^ Its combination of moderate hydrophilicity and high chemical reactivity makes it suitable for advanced applications, including protective coatings,^69^ tissue engineering,^70,71^ and drug delivery systems.^72,73^ Crucially, however, PGMA alone suffers from limited mechanical strength and thermal stability. Therefore, blending PGMA with PVDF provides a means to create a synergistic material that combines the electroactivity and mechanical robustness of PVDF with the functional reactivity and enhanced hydrophilicity of PGMA.^74^

A review of the existing literature reveals that efforts to modify PVDF with PGMA have primarily focused on grafting approaches rather than simple physical blending.^75–77^ Crucially, no systematic study has yet explored the miscibility behavior, phase separation thermodynamics, or resulting morphology of physically blended PVDF/PGMA systems, particularly in the membrane form.

This research, therefore, provides the first comprehensive investigation into the PVDF/PGMA blend system. Our primary goal was to find the miscibility range and develop PVDF/PGMA polymer blend membranes with enhanced functional properties for advanced applications. Our findings establish complete miscibility up to ca. 37 vol % PGMA content, demonstrate that a blend containing 10 vol % PGMA achieves a maximum β-phase content of 70%, and confirm the ability to modulate the membrane porosity and hydrophilicity by adjusting blend composition. This study not only offers essential thermodynamic and morphological insight into this novel blend but also paves the way for the rational design of multifunctional membranes tailored for applications ranging from industrial separation to advanced biomedicine.

## 2 Experimental Section

### 2.1 Materials Description

Polyvinylidene Fluoride (PVDF) was commercially obtained from Alfa Aesar, USA (CAS number: 24937-79-9), having a melt viscosity of 26.22 kilopoise, the molecular weight of which was ${\mathrm{M̅}}_{v}≅{\mathrm{M̅}}_{w}=$ (2.64 ± 0.29)×10^5^, determined by intrinsic viscosity measurements in dilute *N*,*N*-dimethylacetamide (DMAc) solutions at 25 °C, described elsewhere.^78^ The water used had a TDS below 3 ppm, obtained using a locally assembled multistage filtration system combining reverse osmosis and ion exchange. Glycidyl methacrylate (GMA) was purchased from Sigma-Aldrich Co., 3050, Germany. All other chemicals were analytical grade and used as received.

### 2.2 Synthesis and Characterization of PGMA

Poly­(glycidyl methacrylate) (PGMA) was synthesized by free-radical solution polymerization of the monomer glycidyl methacrylate (GMA) utilizing azobis­(isobutyronitrile) (AIBN) as the initiator. In a typical procedure, 0.021 g AIBN was dissolved in 20 mL THF in a 100 mL round-bottom flask by stirring magnetically. 2.084 g GMA was added to the flask and stirred. The system was degassed by bubbling dry nitrogen through the solution for 40 min with the help of a silicone rubber septum arrangement. The free radical polymerization was conducted for 3 h under a nitrogen atmosphere by immersing the flask in a thermostat oil bath at 60 °C. The polymer was recovered from the reaction mixture by precipitating into a large excess of cold methanol. The product was washed five times by centrifugation, decantation, redispersion in THF, and reprecipitation into cold methanol cycles. PGMA was obtained by drying at 50 °C for 24 h under vacuum as a solid (0.735 g, yield: 35%). The polymer was characterized by Fourier transform infrared (FTIR) spectroscopy, gel permeation chromatography (GPC), Intrinsic Viscosity, and differential scanning calorimetry (DSC).

The FTIR (the instrument description in § 2.4.2) absorption peak observed at 902 cm^–1^ was attributed to the epoxide ring stretching (asymmetric C–O–C stretching) vibration in the glycidyl methacrylate (GMA) units of the polymer.^79^ The sharp peak in the spectra at 753 cm^–1^ corresponded to the asymmetric stretching vibration of the epoxy groups,^80^ while the stretching vibration of the carbonyl (C=O) was detected at 1722 cm^–1^.^80,81^

The molecular weight and polydispersity of the synthesized PGMA were determined using a Crozen GPC system (Young In Chromas, South Korea), calibrated using narrow molecular weight polystyrene standards. Tetrahydrofuran THF was employed as the mobile phase at a flow rate of 1.0 mL/min and a column temperature of 35 °C. The resulting chromatogram is displayed in Figure S1, and the molecular weight data are presented in Table S1. The PGMA exhibited a weight-average molecular weight ( ${\mathrm{M̅}}_{w}$ ) of 108.8 kDa, and a polydispersity index ( $PDI={\mathrm{M̅}}_{w}$ / ${\mathrm{M̅}}_{n}$ ) of 1.36.

The intrinsic viscosity ([η]) of the synthesized PGMA was independently measured in THF at 30 °C using an Ostwald viscometer. Detailed experimental and calculation procedures, including the specific Mark–Houwink parameters utilized, are presented in the Supporting Information (Section S2). The obtained intrinsic viscosity yielded a viscosity-average molecular weight ( ${\mathrm{M̅}}_{v}$ ) of 97.2 ± 11.0 kDa. This value is in excellent agreement with the ${\mathrm{M̅}}_{v}$ of 103.7 kDa determined via GPC, substantiating the molecular weight analysis.

The DSC heating thermogram of the polymer (melt-quenched from 200 °C, scanned from 0 to 200 °C at a heating rate of 20 °C/min) exhibited a glass transition (*T*_g_) onset temperature of 68.0 °C.

### 2.3 Preparation of Blend Membranes

The membranes were prepared by a nonsolvent-induced phase separation (NIPS) method from a 10% weight per volume (10% w/v) dope solution of PVDF and PGMA in DMAc. To achieve a homogeneous dope solution, a calculated amount of PVDF + PGMA (10% w/v) was added to DMAc, and the mixture was stirred for 24 h at ambient temperature (25 °C) at 300 rpm. The dope solution was placed in an ultrasonic bath operating in the degassing mode at 60 °C to remove air bubbles. Subsequently, the dope solution was cast onto spotless glass slides by a film applicator with a 300 μm gap. The film was then immersed in a coagulation bath containing a 43.5% by volume (43 vol %) solution of isopropanol in ultrapure water for 10 min at ambient temperature. The membrane-containing slide was then placed in an ultrapure water bath for 24 h, changing the water 2 to 3 times to remove the DMAc from the membrane. The membranes obtained were dried in a vacuum oven at 50 °C. The Compositions and Designations of the prepared membrane samples are presented in Table 2.

### 2.4 Physicochemical Characterization

#### 2.4.1 X-ray Diffraction

X-ray diffraction (XRD) measurement of the films was carried out using a Rigaku Smart Lab-SE diffractometer. The radiation source (Cu K_α_ X-ray, λ = 0.1541 nm) was operated at 40 kV and 40 mA. The scanning was performed from 5° to 80° (2θ) at a scanning velocity of 10°/min. Peak analysis, including baseline correction (using asymmetric least-squares smoothing) and data smoothing (at 60-point windows) was performed using OriginPro 2018 (64 bit) software.

#### 2.4.2 Fourier Transform Infrared Spectrometry

The specific interaction between functional groups of the component polymers and the crystalline phase composition of the PVDF in NIPS-formed PVDF/PGMA blend membranes and melt-treated blends was determined by Fourier transform infrared (FTIR) spectral analysis, using a PerkinElmer infrared spectrometer model FTIR-100. The melt-treatment comprised heating the NIPS-formed blend membranes in a vacuum oven to 200 °C, equilibrating at that temperature for 2 min, and then cooling to ambient temperature under vacuum conditions. The standard method of Gregorio and Cestari^82^ adopted to address the superposition of PGMA absorption bands on the characteristic PVDF bands in the blends, was utilized to determine the total polar polymorph fractions (β and γ) present in PVDF–PGMA blend membranes,^29^ the details of which are given in Supporting Information (Section S4). The determination of the phase composition of PVDF in the samples was crucial for estimating the crystallinity of PVDF and the miscibility of the polymers in the melt state, analyzed by DSC (§ 2.4.3).

#### 2.4.3 Differential Scanning Calorimetry

Thermal transitions of the NIPS-formed blend membranes were examined by a PerkinElmer DSC-8000 (USA) instrument equipped with an Intracooler-II cooling system attached to the differential scanning calorimetry (DSC) furnace. The samples (∼1.0 to 2.0 mg) were sealed in standard aluminum pans, and the scans were performed under the flow (20 mL/min) of dry N_2_ gas from −65 to 200 °C at a heating rate of 20 °C/min. To specifically investigate the melt-state miscibility of the PVDF/PGMA blends (via melting point depression and χ_12_ calculation), NIPS-formed blend samples were melted at 200 °C and then quenched to −65 °C at approximately 150 °C/min. These samples are referred to as melt-quenched blends. The melt-quenched samples were subsequently reheated to 200 °C at the same heating rate (20 °C/min), followed by a final cooling cycle from 200 °C to −65 °C at 10 °C/min. The degree of crystallinity (λ_c_) for both NIPS-formed and melt-quenched blends was calculated from DSC thermograms^83,84^ by applying eq 1.

1 $$\lambda_{c}\;\left(\%\right)=\frac{{\Delta H}_{f}/\varphi }{{\Delta H}_{f}^{*}}\times 100$$

where Δ*H*_f_ is the enthalpy of fusion of the DSC samples, and Δ*H*_f_^*^ is the standard enthalpy of fusion of PVDF, which depends on the relative percentage of various polymorphs of PVDF, and φ is the weight fraction of PVDF in blends.

The fusion enthalpy of 100% pure α polymorph of PVDF was obtained from the literature to be Δ*H*_α_^0^ = 104.5 J/g, and that for 100% pure β-polymorph, Δ*H*_β_^0^ = 219.7 J/g, which was used for the calculation of the combined β- and γ-polymorphs (i.e., as Δ*H*_β+γ_^0^). With this value, the standard enthalpy of fusion^84^ was calculated utilizing eq 2.

2 $${\Delta H}_{f}^{*}={\Delta H}_{\alpha }^{0}\cdot X_{\alpha }+{\Delta H}_{\beta +\gamma }^{0}\cdot X_{\beta +\gamma }$$

where *X*_α_ is the fraction of α polymorph and *X*_β+γ_ is the fraction of (β+γ)-polymorph in a sample. *X*_α_ and *X*_β+γ_ were determined from FTIR analysis of the samples (§ 2.4.2).

For applying the Hoffman–Weeks extrapolation method^85^ for determining the equilibrium melting temperature, the sample was first heated to 190 °C, well above its melting point, to eliminate previous thermal history. After equilibration for 10 min at 190 °C, the sample was quenched to the isothermal crystallization temperatures (*T*_c_) ranging between 128 and 154 °C, with 2 °C increments between each step. After isothermal crystallization at the *T*_c_ for a predetermined period to allow sufficient crystal formation, the sample was reheated in the DSC to record the corresponding melting temperature (*T*_m_). The obtained *T*_m_ values were then plotted against their respective *T*_c_ values according to the following equation.^33,51^

3 $$T_{m}=T_{m}^{0}\left(1-\frac{1}{\gamma }\right)+\frac{T_{c}}{\gamma }$$

here, γ is the thickening ratio. A linear fit of the plot of *T*_m_ versus *T*_c_ according to this equation was extrapolated to its intersection with the line *T*_m_ = *T*_c_, and the intersection point was taken as the equilibrium melting temperature (*T*_m_^0^).

The polymer–polymer interaction parameter (χ_12_) was calculated using the expression proposed by Nishi and Wang,^29^ details of which are presented in the discussion section (*cf* § 3.5.6).

#### 2.4.4 Thermogravimetric Analysis

The thermal stability of the blend membranes was determined by thermogravimetry using a PerkinElmer STA-6000, a simultaneous TG/DTA Instruments. The thermogram was recorded in the temperature range of 30 to 800 °C at a scan rate of 10 °C/min under a nitrogen gas flow.

#### 2.4.5 Membrane Morphological Quantification by FESEM

The membrane films were first fractured by freezing in liquid nitrogen and dried in a vacuum. They were then sputter-coated with gold to observe their surface and cross-sectional morphology by a JEOL (Japan) JSM IT800 field emission scanning electron microscope (FESEM), operated at an accelerating voltage of 15 kV.

The cross-sectional FESEM images were analyzed using ImageJ software for estimating critical structural dimensions, specifically membrane thickness and pore size (diameter). For the free-standing membranes (*vide* Figure S4 of the Supporting Information) tested, the membrane thickness ranged between 23 and 47 μm, whereas the pore size ranged between 0.20 and 3.4 μm. The comprehensive membrane thickness and pore size data, including error estimates, have been presented in Table S3, and the detailed pore size distribution curves have been displayed in Figure S3.

#### 2.4.6 Membrane Porosity

The porosity of the membranes was determined gravimetrically.^78^ The membranes were chopped into small pieces and were weighed individually in a semimicro analytical balance. Then each piece of membrane was immersed in heptanol for 2 min. The excess solvent on the surface of the membrane was sucked by filter paper and weighed. The porosity of the membrane was calculated using eq 4.^86,87^

4 $$porosity,e=\frac{\frac{{w_{2}-w}_{1}}{\rho_{a}}}{\left(\frac{w_{2}{-w}_{1}}{\rho_{a}}+\frac{w_{1}}{\rho_{m}}\right)}\times 100$$

Where *w*_1_ is the mass of the dry membrane, *w*_2_ is the mass of the wet membrane, ρ_a_ is the density of the alcohol, and ρ_m_ is the density of the membrane material.

#### 2.4.7 Membrane Hydrophilicity

The hydrophilicity of the NIPS-formed blend membrane surfaces was evaluated by measuring the static water contact angle (WCA) using a contact angle goniometer (model L2004A1, Ossila Ltd., Sheffield, England). The measurements were performed via the sessile drop method, where a deionized water droplet was gently placed on the membrane surface, and its profile at the solid–liquid–air interface was captured from the side. The contact angle was then calculated by the instrument’s software by curve-fitting the droplet profile. At least three measurements were conducted at different locations for each membrane sample to ensure reliability, and the average value was reported.

## 3 Results and Discussion

### 3.1 Assessment of PVDF/PGMA Miscibility: A Thermodynamic Perspective

Homogeneous polymer blends result from thermodynamically favorable mixing, which is indicated by a negative Gibbs free energy of mixing (Δ*G*_M_) and a positive second derivative of the free energy across all compositions,^88,89^ i.e.

5 $$\Delta G_{M}<0$$

6 $${\left(\frac{\partial^{2}\Delta G_{M}}{\partial \varphi_{i}^{2}}\right)}_{T,P}>0$$

While the second derivative is crucial for identifying stable as well as metastable regions of compatibility, the primary focus on determining blend miscibility often lies in Δ*G*_M_

7 $$\Delta G_{M}=\Delta H_{M}-T\Delta S_{M}$$

For polymer blends, the entropic contribution (Δ*S*_M_) to free energy of mixing is typically negligible. Therefore, a negative enthalpy of mixing (Δ*H*_M_), indicating specific attractive interactions, can be a reasonable proxy for predicting blend miscibility.

The Schneier equation^90^ offers a practical tool for predicting the miscibility of binary polymer blends.^45^ It estimates the heat of mixing (Δ*H*_M_) for two polymers based on differences in their solubility parameters, on the concept that the heat of mixing (Δ*H*_M_) is related to the differences in their solubility parameters and volumetric properties^90^

8 $$\Delta H_{M}={\left[x_{1}M_{1}\rho_{1}{\left(\delta_{1}-\delta_{2}\right)}^{2}\times {\left\{\frac{x_{2}}{x_{1}M_{2}\rho_{2}+x_{2}M_{1}\rho_{1}}\right\}}^{2}\right]}^{1/2}$$

Here, subscripts 1 and 2 represent PVDF and PGMA, respectively, while *x*_*i*_,*M*_*i*_, ρ_*i*_ and δ_*i*_ represent the weight fraction, molecular weight of the repeating unit, the density, and the solubility parameter of the corresponding polymer. The solubility parameter measures a polymer’s cohesive energy density, and a smaller difference (δ_1_–δ_2_), generally indicates greater compatibility. For a system to be miscible, Δ*H*_M_ should ideally be negative or close to zero. Schneier suggested a threshold value of 0.04184 J/mol (of 0.01 cal/mol) for evaluating compatibility: a value below this threshold implies complete miscibility, while exceeding it indicates partial miscibility or complete immiscibility.^90^

To validate the applicability of the Schneier equation, we re-evaluated published data for several binary polymer blends involving PVDF. For instance, Aid et al. reported experimental Flory–Huggins interaction parameter (χ_12_) for the PVDF/PMMA system as – 0.56, −0.54, and −0.26 for blends with 10%, 30%, and 90% PMMA content, respectively, indicative of thermodynamic stability and complete miscibility.^29^ Our calculations of Δ*H*_M_ according to the Schneier equation for these compositions consistently fall below the theoretical threshold across the entire composition range (Figure 1), validating the Schneier equation for PVDF/PMMA blends. A similar analysis of the PVDF/PBA system also indicates complete miscibility (Figure 1), consistent with the experimental observations by Penning et al.^52^ Furthermore, for PVDF/PEO blends, where the Flory–Huggins interaction parameter suggests miscibility, particularly in PVDF-rich compositions, the Schneier equation accurately predicts a miscibility gap of up to 78 vol % PVDF content (Figure 1).^47^

**Figure 1:**
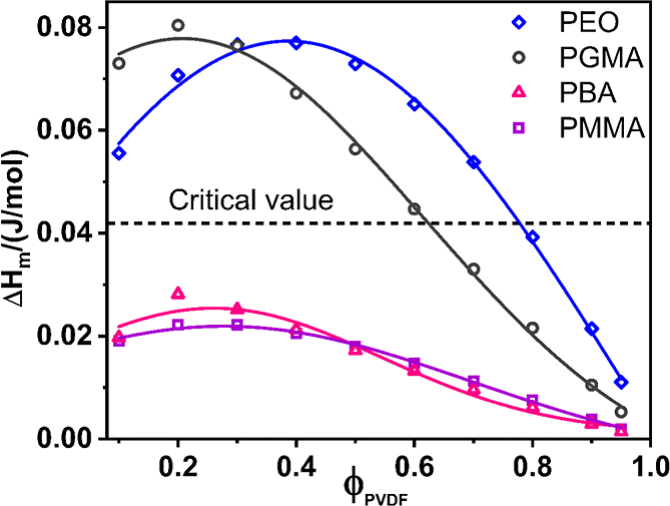
Calculated enthalpy of mixing (Δ*H*_M_) for various PVDF blends. The enthalpy of mixing, calculated using the Schneier equation, is plotted as a function of the PVDF volume fraction (ϕ_PVDF_) for the PVDF/PMMA, PVDF/PEO, PVDF/PBA, and PVDF/PGMA blend systems, as indicated by the legend.

Having validated its utility, we applied the Schneier equation to predict the miscibility of PVDF/PGMA blends. Utilizing literature values for the solubility parameters of PVDF (23.2 MPa^1/2^)^86^ and PGMA (25.8 MPa^1/2^),^91^ we calculated the theoretical Δ*H*_M_ across the entire composition range. The results are presented in Figure 1, where a dotted horizontal line indicates the critical mixing enthalpy. Based on these theoretical calculations, a PGMA volume fraction of 37% in the blend corresponds to the threshold Δ*H*_M_ value, predicting that blend compositions below this critical PGMA content are theoretically miscible, while those with higher PGMA content are supposed to undergo phase separation.

It is important to note that the Schneier equation provides an approximation, and its derived Δ*H*_M_ values offer a general indication rather than a definitive measure of miscibility. Therefore, this threshold blend composition should serve as a preliminary guideline only, as polymer miscibility is a complex phenomenon influenced by multiple factors beyond those considered in this equation. Consequently, we rigorously evaluated the miscibility of PVDF/PGMA blends using a comprehensive suite of experimental and thermodynamic techniques, including analysis of peak shifts in FTIR and XRD, observation of phase segregation in FESEM micrographs, melting point depression measurements by DSC, and direct Flory–Huggins interaction parameter (χ_12_) calculation.

### 3.2 Microstructure of PVDF in Blend Membranes: XRD Analysis

Figure 2 displays the X-ray diffraction patterns of the NIPS-formed PVDF/PGMA blend membranes. The diffraction pattern of the neat PGMA membrane was flat, confirming its amorphous nature. Conversely, the broad diffraction peaks of PVDF in the blend membranes reflect their low crystallinity, which is typically <30% (*cf*. DSC data in Table 3). This low crystallinity, inherent to NIPS-formed polymeric membranes, makes a detailed quantitative analysis of crystal structure from XRD data less reliable. For this reason, we have relied on DSC for the quantitative degree of crystallinity values.

**Figure 2:**
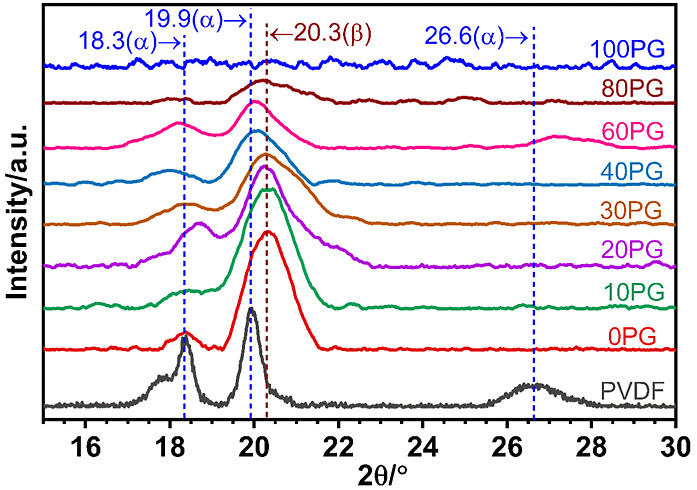
Wide-angle X-ray diffraction (XRD) patterns of PVDF/PGMA blend materials: XRD patterns of neat PVDF powder, NIPS-formed pure PVDF membrane (0PG), the series of PVDF–PGMA blend membranes (5PG—80PG), and the pure PGMA membrane (100PG).

**Table 3:** Structural and Thermal Properties of NIPS-Formed PVDF/PGMA Blend Membranes: Lamellar Thickness (*L* spacing) from XRD, Polar Phase Fraction (β+γ) from FTIR, and Quantitative DSC Results^b^

sample name	(*L* ± δ*L*)/nm	(β+γ)-phase (±0.7)/%	*T*_g_ (onset) (±0.1)/°C	*T*_m_ (peak) (±0.1)/°C	normalized *H*_m_ ((±0.1)/J/g)	(λ_c_ ± δλ_c_)^a^/(%)
PVDF	17.7 ± 0.1	17.2	—	160.0	32.2	25.9 ± 0.3
0PG	7.7 ± 0.1	62.6	—	158.3	55.3	31.3 ± 0.2
5PG	5.9 ± 0.5	68.0	—	156.8	48.4	26.5 ± 0.2
10G	6.7 ± 0.4	69.8	—	157.4	50.5	27.3 ± 0.2
20PG	5.5 ± 0.3	59.3	63.7	157.5	48.6	28.1 ± 0.2
30PG	4.8 ± 0.6	57.3	—	157.1	41.1	24.1 ± 0.2
40PG	7.2 ± 0.3	50.3	—	158.0	44.1	27.1 ± 0.2
50PG	6.7 ± 0.6	47.6	51.2	158.2	52.9	33.2 ± 0.2
60PG	7.6 ± 0.7	56.9	—	156.8	55.1	32.4 ± 0.2
80PG	5.7 ± 0.5	55.8	80.6	154.9	44.9	26.6 ± 0.2
100PG		—	81.1	—	—	—

a The crystallinity (λ_c_) was calculated from the DSC enthalpy of fusion, factoring in the (β+γ)-phase fraction determined by FTIR, due to the difference in melting enthalpy between the polar (β,γ) and nonpolar (α) polymorphs, using the Gregorio and Cestari method.^82^

b DSC data for the as-prepared neat PGMA were unreliable due to thermal artifacts and are therefore not reported.

Nevertheless, XRD analysis provides valuable qualitative insights. For a detailed record, the interplanar distance (*d*-spacing) and lamellar thickness (*L*-spacing) analyses are available in the Supporting Information (Section S3). This analysis revealed a nonmonotonic variation of L-spacing with blend composition that conspicuously mirrors the DSC melting data. This trend, which is consistent with the miscibility gap predicted by the Schneier equation (discussed in § 3.1), indicates that the subtle changes in crystallite size and morphology directly result from the blending-induced miscibility and phase separation.

However, the relative intensity and position of the diffraction peaks clearly demonstrated the effect of blending on the microstructure of the crystals formed in the membranes. The commercial PVDF powder displayed characteristic diffraction peaks at 2θ = 19.9° (110), 18.3° (020), and 26.6° (021), confirming its predominant crystalline α-phase with a monoclinic unit cell.^92,93^ However, in the neat PVDF membrane, while the α-phase (020) peak at 18.3° drastically diminished, a predominant β-phase (orthorhombic unit cell) peak appeared at 20.26° (110), indicating a significant transformation to the β-phase during membrane processing.^94^ Notably, the diffraction patterns of the blend membranes also exhibited the dominant peak at 20.26° (110), suggesting a substantial presence of the β-phase PVDF within the blends.^94,95^

The molecular compatibility between dipolar PVDF segments and the polar solvent DMAc used in the dope solution was crucial in promoting the β-phase PVDF crystallization in these membranes.^78,94^ Specifically, the high polarity of the solvent promoted rotation of the dipole moments of the C–F bonds in the PVDF molecular chain, thereby lowering the energy barrier for crystallization into the more extended all-trans (TTTT) β-conformation.^94,96^ Furthermore, a weak hydrogen bonding between the carbonyl group oxygen (>C=**O**) in DMAc and slightly acidic hydrogen atoms in the methylene group next to the CF_2_ dipole (−C**H**_2_–CF_2_−) in PVDF (C=**O**·······**H**–CH) is also possible.^94,97^ The oxygen in the carbonyl group of the diluent polymer PGMA in the dope solution can also interact with the PVDF chain segments in the same fashion as the solvent; both interactions might act synergistically to significantly increase the β-phase PVDF content in the blend membranes, producing the strong diffraction maxima at 2θ = 20.26° characteristic of the β-phase and the very weak diffraction at 2θ = 18.3° and 19.9° associated with the α-phase.

It was also noted that the diffraction band centered at 2θ = 20.26° associated with the β-phase of PVDF significantly broadened and shifted toward lower angles for blends with 40% and higher PGMA content, suggesting a decreased interaction between the two polymer chains within this specific composition range.^98,99^ This finding is further supported by the FTIR analysis discussed in the following section.

### 3.3 Microstructural Interactions and Crystalline Phase Evolution: FTIR Study

#### 3.3.1 Microstructure and Specific Interaction

Figure 3a displays the FTIR spectra of NIPS-formed PVDF/PGMA blend membranes, neat polymer membranes, and the as-received commercial PVDF powder, while Figure 3b depicts the FTIR spectra of the corresponding melt-treated samples. It was noticed from the Figures that the IR absorption features of NIPS-formed blend membranes and the corresponding melt-treated samples were almost identical. The neat PGMA membrane (100PG) displayed a sharp absorption peak characteristic of the carbonyl group (C=O) stretching frequency at 1722 cm^–1^,^80^ and the epoxy ring’s asymmetric stretching vibration frequency at 753 cm^–1^,^80^ and 843 cm^–1^.^81^ In the blend membranes, the absorption band at 753 cm^–1^ of PGMA was superimposed on the band at 764 cm^–1^ associated with the CF_2_ bending vibration of the α-phase PVDF.^78,100^ Likewise, the band at 843 cm^–1^ of PGMA was superimposed on the band at 840 cm^–1^, assigned to the combined β-phase and the γ-phase of PVDF in the blends.^78^ However, the sharp peak at 902 cm^–1^, attributed to the asymmetric C–O–C stretching vibration of the oxirane (epoxy) ring in GMA units,^79^ retained its position in the blend membranes, confirming the preservation of the epoxy ring structure throughout the PGMA polymerization and subsequent membrane formation protocols via blending.

**Figure 3:**
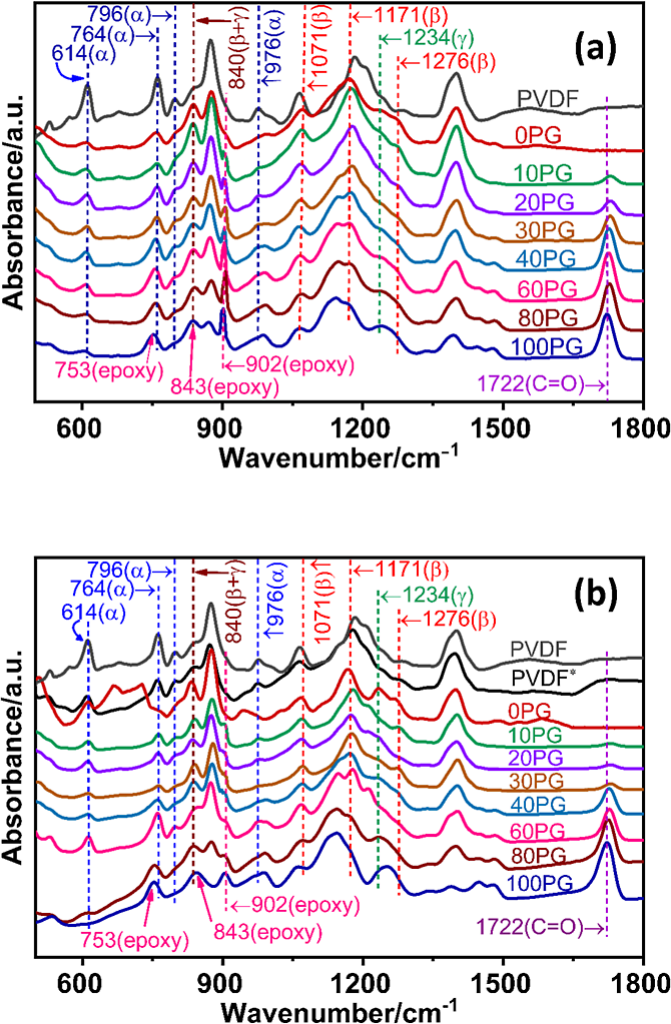
Representative FTIR spectra of: (a) NIPS-formed membranes of PVDF/PGMA blends and neat polymers; (b) corresponding melt-treated samples (heated to 200 °C and cooled to ambient). In the legends, PVDF and PVDF* represent the as-received neat polymer, and the melt-treated PVDF, respectively; while xPG indicates *x* vol % PGMA content in a blend. Note: the NIPS-formed membrane samples (0PG–100PG) exhibited almost identical FTIR spectra after melt treatment.

In the FTIR spectra of PVDF powder, relatively sharp and intense peaks recorded at 614, 764, 796, and 976 cm^–1^ illustrated that it contained predominantly the α-phase crystals.^92^ However, in the NIPS-formed neat and blend membranes, the intensity of these peaks decreased significantly. In contrast, absorption peaks characteristic of the β-phase PVDF at 1071, 1171, and a shoulder at 1276 cm^–1^ evolved with significant intensity. The absorption band at 840 cm^–1^ attributed to both β and γ phases, results from the combined absorption for the bending of CH_2_ and the unbalanced stretching movement of CF_2_.^100^ A sharp and well-resolved peak in this position represents pure β-phase, while a broad band represents pure γ-phase.^92,94,101,102^ The spectrum of the commercial PVDF powder displayed a broad shoulder band at 840 cm^–1^, primarily indicating the presence of the γ-phase.^78^ In sharp contrast, all NIPS-formed membranes exhibited a sharp, well-resolved absorption peak centered at 840 cm^–1^. This transformation confirms the effective induction of the electroactive β-phase PVDF during the NIPS process, a conclusion that is consistent with the findings of the XRD analysis.^94,103^

For both the NIPS-formed and the melt-treated blends, the carbonyl group (C=O) stretching frequency of PGMA suffered a substantial (10 cm^–1^) higher frequency shift for the lowest PGMA content blend (5PG) from the original 1722 cm^–1^ of 100PG, which, however, gradually reduced with increasing PGMA volume fraction in the blends. This shift is a strong indication of the presence of dipolar interaction between the carbonyl group of PGMA and the −CF_2_– dipole of PVDF, the extent of which is most prominent in the low PGMA content blends.^29^ This spectroscopic evidence qualitatively supports the theoretical predictions from the Schneier equation, which estimates favorable miscibility for PVDF/PGMA blends at low PGMA content (up to approximately 37 vol % PGMA).

#### 3.3.2 PVDF Crystalline Phase Composition of Blends

The phase composition of the PVDF component in the blend membranes was quantitatively determined by FTIR spectroscopy by adopting the standard method of Gregorio and Cestari^82^ to address the superposition of PGMA absorption bands at 753 cm^–1^ and 843 cm^–1^ on the characteristic PVDF peaks of 764 cm^–1^ (α) and 840 cm^–1^(β), respectively. The fraction of the polar β & γ phases in crystalline PVDF in NIPS-formed membranes and in melt blends are presented in Tables 3 and 4, respectively. Details of the calculation are available in the Supporting Information (Section S4).

**Table 4:** Summary of Thermal Properties from DSC Analysis of Melt-Quenched and Melted PVDF/PGMA Blends

sample name	(β+γ)-phase (±0.7)/%	DSC Heating^a^				DSC Cooling^b^	
		(*T*_g_ ± 0.1)/°C	(*T*_m_ ± 0.1) /°C	(Δ*H*_m_ ± 0.1)^c^/(J/g)	(λ ± δλ)^d^/%	(*T*_c_ ± 0.1) /°C	(Δ*H*_c_ ± 0.1)^c^/(J/g)
PVDF	28.7	—	160.1	34.8	25.3 ± 0.3	131.2	–34.4
0PG	64.7	—	160.9	43.3	24.2 ± 0.2	134.6	–41.8
5PG	75.1	—	159.6	39.9	20.9 ± 0.3	131.2	–40.5
10PG	69.2	—	159.2	38.7	21.0 ± 0.3	132.2	–38.8
20PG	61.4	—	158.6	38.2	21.8 ± 0.3	128.1	–40.4
30PG	70.1	—	157.7	36.8	19.9 ± 0.3	127.3	–40.4
40PG	66.6	—	157.6	39.9	22.0 ± 0.3	123.6	–41.6
50PG	63.2	—	158.0	44.1	24.9 ± 0.2	123.2	–45.7
60PG	49.2	15.1	155.2	48.7	30.2 ± 0.2	118.8	–49.0
80PG	29.4	—	149.5	29.9	21.6 ± 0.3	107.0	–21.3
100PG	—	62.2	—	—	—	—	—
PGMA	—	68.0	—	—	—	—	—

a Heating was performed at 20 °C/min after quenching the samples from 200 °C to −65 °C.

b Subsequent cooling of the melted samples was performed at 10 °C/min.

c Enthalpy values (Δ*H*_m_ and Δ*H*_c_) were normalized to the mass fraction of PVDF in the blend.

d Crystallinity was calculated based on the (β+γ)-phase fractions, estimated using the Gregorio and Cestari method^82^ on the FTIR spectra of the melted samples.

The as-received PVDF powder exhibited 17.2 vol % polar (β+γ) phase content (*vide* Table 3). On the contrary, the NIPS-formed neat PVDF (0PG) membrane displayed a dramatic increase in the polar phase content to 62.6%. In PVDF/PGMA blend membranes, the (β+γ) phase content initially rose further with increasing PGMA content, peaking at 69.8% for the 10PG, beyond which it gradually decreased through midrange PGMA concentrations (viz., to 47.6% at 50PG), before showing a slight recovery (∼56%) at higher PGMA loadings. This substantial induction of polar phases by the NIPS process, especially in the presence of even small amounts of PGMA, highlights its profound role in influencing PVDF crystallization pathways.

Upon melt-treating the neat PVDF powder, the polar phase content increased to 28.7 vol %, an enhancement of 67% compared to the as-received polymer. This initial increase confirms that rapid cooling alone promotes the formation of these polar phases.

However, the melt-treated blend membranes demonstrated a much higher performance baseline, beginning at 64.7% for melt-treated neat PVDF (0PG). The polar phase content reached an even higher maximum of 75.1% at a very low (5 vol %) PGMA content. The polar-phase fraction remained high across midrange PGMA compositions (e.g., 70.1% for 30PG, 66.6% for 40PG), before undergoing a strikingly steep decrease at higher PGMA concentrations, plummeting to 49.2% for 60PG and dropping drastically to 29.4% for 80PG blend, which is a −14% and −47% decrease, respectively, in polar phase content relative to their NIPS-formed counterparts.

The comparison of processing routes highlights a complex interplay of miscibility and kinetics. Melt-treatment generally increased the polar-phase content in the low-to-mid PGMA range, confirming that rapid thermal quenching further stabilizes the polar PVDF conformation within the relatively homogeneous melt. On the contrary, melt-treatment of the NIPS-formed high-PGMA blends (60PG and 80PG) dramatically reduced the polar-phase content. This divergence can be attributed to the initial PVDF chain conformation under the two processing protocols: In the NIPS process, the pre-extended PVDF chains in the dope solution, resulting from the dipolar interaction with the solvent (DMAc), retain their conformation during solidification, promoting the polar β/γ phases even in phase-separated domains. On the contrary, in the highly diluted (high-PGMA) melt state, PVDF chains in phase-separated domains are in a random coil conformation. Rapid quenching of these isolated, unconstrained domains kinetically favors the nonpolar α-phase, leading to the sharp drop in polar-phase content.

### 3.4 Thermal Stability and Degradation Behavior: TGA Analysis

Representative TGA thermograms shown in Figure 4a, and their corresponding derivative thermogravimetry (DTG) profiles shown in Figure 4b of the system, reveal that PVDF (both powder and membrane form) had superior thermal stability compared to PGMA, which underwent a two-step degradation. However, all the blend membranes were thermally stable up to 250 °C, which confirmed their safe manipulation within this temperature range for the DSC study, discussed in the next section.

**Figure 4:**
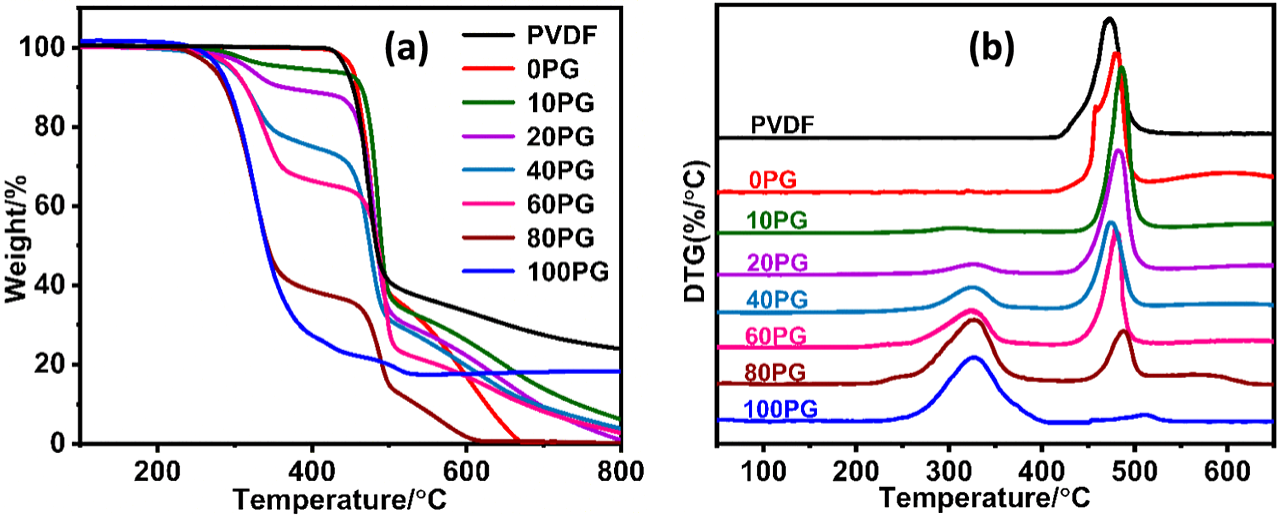
TGA thermograms (a) and DTG profiles (b) of neat PVDF powder and the NIPS-formed membranes: neat PVDF (0PG), neat PGMA (100PG), and PVDF/PGMA blends of varying composition (*x*PG in the legend indicates *x* vol % PGMA content).

A notable observation is the difference in residual mass: the neat PVDF membrane (0PG) degraded almost entirely below 700 °C, while the commercial PVDF powder exhibited a significant char yield of 24% even at 800 °C. Despite the expected identical degradation chemistry, this discrepancy is attributed to the NIPS fabrication process. The rapid phase inversion likely created a distinct membrane morphology (e.g., higher porosity, less crystal perfection, altered chain packing, or potential trace residuals from processing aids) that kinetically or thermodynamically favored more complete volatilization of degradation products compared to the bulk powder, which exhibited a more stable char-forming pathway.

The DTG curves clearly show that all blends underwent two-step degradation, with peaks corresponding to both PVDF and PGMA components. As PGMA content increased, the low-temperature peak of the DTG profile became more pronounced, while the high-temperature peak due to PVDF diminished, directly reflecting compositional changes.

### 3.5 Melting, Crystallization, and Miscibility of PVDF/PGMA Blends: DSC Study

#### 3.5.1 Effect of PGMA Miscibility on the Melting of PVDF in NIPS-Blend Membranes

Figure 5a displays the DSC heating thermograms of the as-received PVDF powder, NIPS-formed neat PVDF, and PVDF/PGMA blend membranes, with corresponding data presented in Table 3. The *T*_g_ of the NIPS-blend membrane samples was uncertain and irregular, rendering them unsuitable for concluding polymer miscibility. However, the melting temperature (*T*_m_) of PVDF, presented as a function of the blend composition in Figure 5b, proved to be a valuable indicator.^59^ The as-received PVDF powder showed a *T*_m_ of 160.0 °C. In contrast, the NIPS-formed PVDF membrane (0PG) exhibited a *T*_m_ of 158.3 °C, presenting a 1.7 °C depression from the powder. This depression is notably supported by a significant decrease in lamellar thickness (*L*-spacing) from 17.7 nm for the powder to 7.7 nm for the 0PG membrane (Table 3) in XRD data analysis.

**Figure 5:**
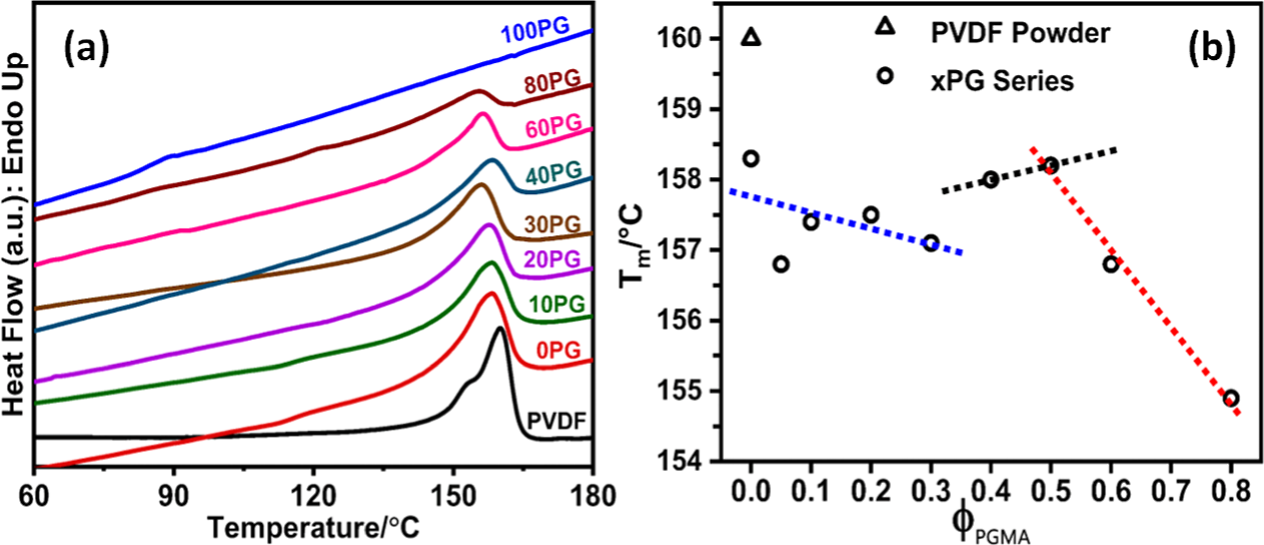
Thermal analysis of PVDF/PGMA blend membranes. (a) Representative DSC heating thermograms showing the melting behavior of the neat PVDF powder and the NIPS-formed PVDF/PGMA blend membranes. (Experimental conditions: samples were cooled to −65 °C and heated to 200 °C at the rate of 20 °C/min under a N_2_ atmosphere.) (b) Plot of the observed melting temperatures (*T*_m_) as a function of the PGMA volume fraction. The dotted lines serve only as a guide to the eye.

We may note here that the observed melting point (*T*_m_) of a polymer crystallized under ordinary conditions is a function of the thickness (*L*) of the lamellar crystal formed, among other factors, as given by the Gibbs–Thomson equation^104^

9 $$T_{m}=T_{m}^{0}\left(1-\frac{2\sigma_{e}}{\Delta H_{f}L}\right)$$

where *T*_m_^0^ is the equilibrium melting temperature of an infinitely thick crystal, σ_e_ is the surface free energy of the basal plane of the lamella, Δ*H*_f_ is the enthalpy of fusion per unit volume. This equation clearly shows that as *L* decreases, *T*_m_ is depressed below *T*_m_^0^. This is because smaller crystals have a higher surface area to volume ratio, making them thermodynamically less stable and hence, easier to melt.

For the NIPS-blend membranes, the *T*_m_ of PVDF displayed a complex, multistage trend as a function of PGMA content (*cf*. Figure 5b). Initially, *T*_m_ decreased from 158.3 °C for 0PG (neat PVDF) membrane to 157.1 °C for 30PG blend membrane with a concurrent decrease in lamellar thickness (*L*) from 7.7 to 4.8 nm, respectively (*vide* Table 3). This consistent decrease in both *T*_m_ and L suggests that the initial introduction of PGMA hinders the crystallization of PVDF into thicker lamellae. This hindrance is likely due to the increasing volume fraction of PGMA disrupting PVDF chain packing during crystallization from the solution phase, even as the system moves toward a less favorable miscibility boundary. Subsequently, *T*_m_ notably jumped to a neat PVDF level (158.3 °C) and plateaued at this temperature up to 50 vol % PGMA concentration. This distinct recovery and plateau in *T*_m_ strongly suggest the onset of macroscopic phase separation within this composition range, where PVDF chains can crystallize within their own segregated domains, experiencing less hindrance from PGMA. Finally, the *T*_m_ continued to decrease further for PGMA concentrations beyond 50 vol %. This renewed depression reflects the combined effects of extreme dilution and increased kinetic confinement of PVDF even within phase-separated domains, forming less-perfect crystals.

The nonmonotonic trend in the DSC melting temperatures suggests the PVDF/PGMA NIPS-blends exhibit partial miscibility. The data establish the miscibility boundary to be located between 30 vol % and 40 vol % PGMA, where phase separation begins to dominate the thermal behavior.

#### 3.5.2 Effect of PGMA Miscibility on the Melting of PVDF in Melt-Quenched Blends

The NIPS-formed PVDF/PGMA blend membranes were subjected to a uniform thermal history in the DSC by quenching the melt from 200 °C to −65 °C at a cooling rate of 150 °C/min. This rapid quenching was performed primarily to reduce crystallization during cooling and, ideally, to reveal the glass transition temperature (*T*_g_). Figure 6a displays the DSC heating thermograms (heating rate 20 °C/min) of these melt-quenched materials. Consistent with observations for the NIPS-blend membranes, the *T*_g_ of the melt-quenched blends also remained uncertain and irregular in the heating thermograms, precluding their use as a reliable indicator of polymer miscibility.^33^ However, the melting points, plotted as a function of the blend composition of these melt-quenched samples, revealed notable differences depending on their initial form. The as-received commercial PVDF powder melted at 160.0 °C, and when this same powder was melt-quenched, it recrystallized and subsequently melted at a very similar temperature (160.1 °C). This indicates that the intrinsic crystallization behavior of the pure PVDF powder is robust and largely unaffected by the melt-quenching process, yielding a consistent lamellar structure that dictates its melting point.

**Figure 6:**
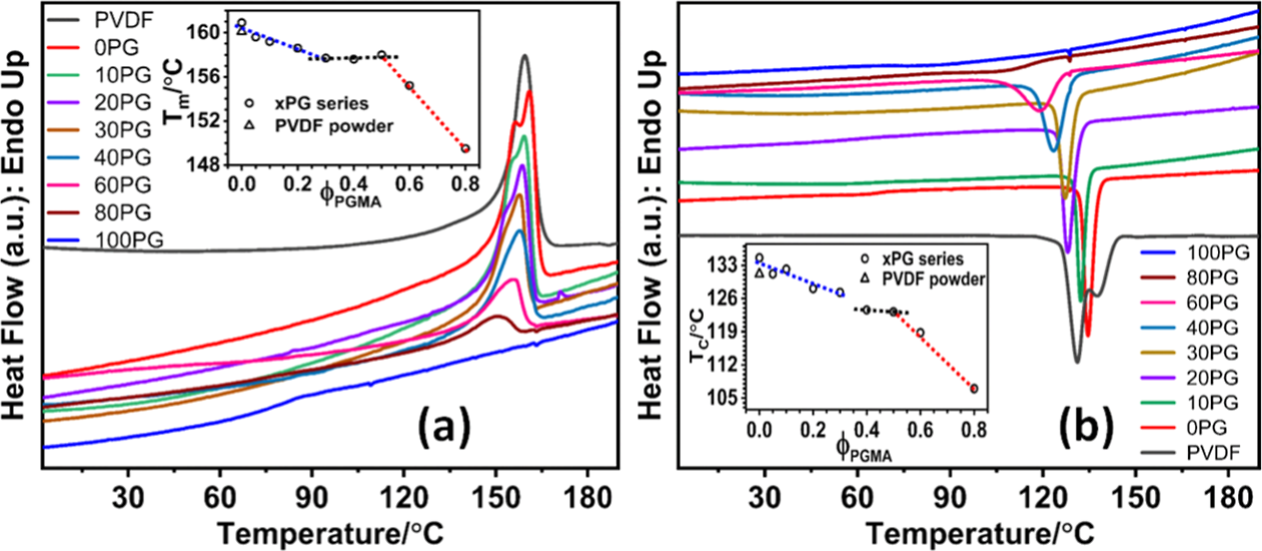
DSC Analysis of Melt-Quenched PVDF/PGMA Blend Materials: (a) DSC Heating thermograms (heating rate 20 °C/min) of melt-quenched blend materials (prepared by cooling from 200 °C to −65 °C). Inset: Plot of corresponding melting temperatures (*T*_m_) of PVDF as a function of the PGMA volume fraction. (b) DSC cooling thermograms (cooling rate: 10 °C/min) of the same samples following the melt history. Inset: Plot of the corresponding dynamic crystallization temperatures (*T*_c_) of PVDF as a function of the PGMA volume fraction. The dotted lines serve only as a guide to the eye.

In contrast, the neat PVDF membrane (0PG) prepared by the NIPS method, which initially melted at a lower temperature of approximately 158.3 °C (about 2 °C lower than the commercial powder, as discussed previously), showed a higher melting temperature of 160.9 °C when subjected to the melt-quenching protocol. This significant observation suggests that the NIPS process initially constrained PVDF crystallization, leading to less perfect or thinner lamellae and thus a lower *T*_m_. However, by melting and then recrystallizing this NIPS-formed PVDF (0PG) in the DSC, the polymer chains reorganized more favorably, overcoming the kinetic limitations imposed by NIPS, and formed more perfect or thicker lamellae that resulted in a *T*_m_ even slightly higher than the original commercial powder. This fact highlights that the NIPS process, rather than the intrinsic PVDF properties, was responsible for the initial *T*_m_ depression in the NIPS-formed neat PVDF membrane.

For the melt-quenched blends, the *T*_m_ of PVDF displayed a complex, multistage trend (*vide* Inset of Figure 6a), remarkably similar to that observed for the NIPS-blend membranes. Specifically, *T*_m_ initially decreased from 160.9 °C (0PG) to 157.7 °C with increasing PGMA content up to 30 vol %. It then plateaued at this lower level with further increase of PGMA to 50 vol %, before finally continuing to sharply decrease at higher PGMA concentrations (a depression of 11.4 °C, at 80PG). This intricate *T*_m_ behavior in melt-quenched samples, directly mirroring the NIPS trend, further solidifies the proposed partial miscibility and subsequent phase separation mechanism in the PVDF/PGMA blend system.

Crucially, for the melt-quenched blends, the initial depression of 3.2 °C in *T*_m_ (up to 30% PGMA) was much higher compared to the corresponding 1.2 °C depression of NIPS-formed blends. This more pronounced *T*_m_ depression in the melt-quenched system indicates a more significant hindrance to PVDF crystallization by PGMA up to 30% in the melt-blends. This difference can be attributed to the fact that in the NIPS-formed blends, the carbonyl group of PGMA had to compete with the carbonyl group of the solvent, DMAc, in the dope solutions for interaction with the (−CF_2_−) dipole along the PVDF chain before the polymers were precipitated in the coagulation bath, thereby reducing the extent of direct PVDF–PGMA interaction. While the calculated Flory–Huggins interaction parameter (χ_12_) values for the system (*vid*e § 3.5.6) indicated that the average PVDF–PGMA attractive interaction strength decreased (χ_12_ became less negative) as PGMA concentration increases within the range 0PG–30PG, the increasing volume fraction of PGMA (higher dilution) nonetheless posed a stronger kinetic hindrance to PVDF chain packing in the melt-blends. This disruption of organized crystallization in the partially miscible melt, especially with stronger initial interaction (at low PGMA), leads to the formation of smaller and less perfect lamellae, as reflected in the observed *T*_m_.

The subsequent plateau (between 30 and 50% PGMA) is consistent with the onset of macroscopic phase separation, where PVDF chains can crystallize within their own distinct domains, experiencing less influence from the increasing PGMA content. Finally, the renewed decrease in *T*_m_ at high PGMA concentrations (beyond 50%) indicates the extreme dilution and kinetic confinement of PVDF. Notably, the 80 vol % PGMA blend exhibited a massive *T*_m_ depression of 11.4 °C (compared to 1.9 °C in the NIPS-formed blend at 80% PGMA). This substantial depression is attributed to the increasing dominance of PGMA in dictating the thermal environment, which severely limits PVDF’s ability to self-assemble into well-developed crystals, even within phase-separated microdomains. The overall multistage trend in melt-quenched *T*_m_ suggests a complex interplay between polymer miscibility, PGMA’s role as a diluent or an interacting component, and the kinetics of crystallization during quenching.

#### 3.5.3 Comparative Crystallization Kinetics of PVDF/PGMA Blends: NIPS vs Melt-Quenching

##### 3.5.3.1 Baseline Crystallinity and Polymorphism (NIPS vs Melt-Quenched)

The degree of PVDF crystallization (λ_c_) quantitatively was determined from the enthalpy of melting (Figure 7, Table 3 and 4), offering insight into the influence of blend composition and processing history on the polymer’s crystalline structure.

**Figure 7:**
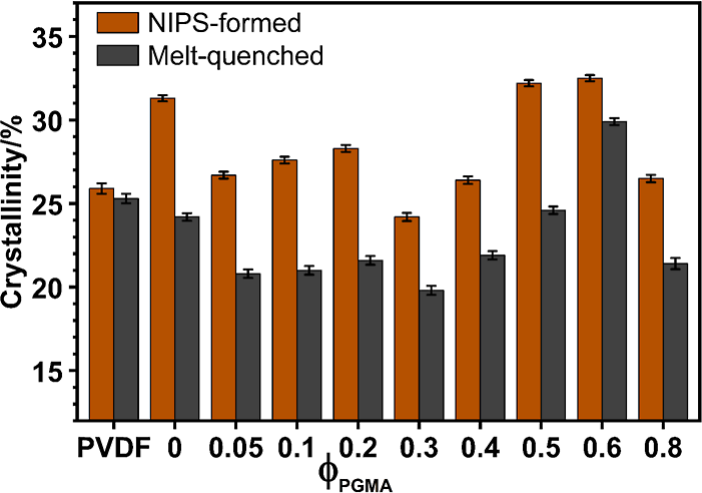
Degree of Crystallinity (λ_c_) of PVDF as a function of PGMA volume fraction (ϕ_PGMA_). The data represent samples prepared via NIPS (nonsolvent-induced phase separation) and melt-quenching, determined from the enthalpy of fusion (Δ*H*_m_) of DSC melting endotherms. The Δ*H*_m_ values were calculated by incorporating the fraction of different PVDF polymorphs, as determined from FTIR data analysis using the method proposed by Gregorio (vide S4 of Supporting Information).

Initially, the NIPS process itself caused a significant enhancement in neat PVDF (0PG) crystallinity, increasing it from 26% (original powder) to approximately 31%. This significant enhancement is attributed to the mechanism of rapid phase inversion inherent to NIPS, where the induced shear stress and fast chain collapse strongly promoted the formation of the polar β-phase (confirmed by FTIR and XRD).^105^ This preferential crystallization into the highly ordered β-phase is the primary driver for the observed increase in the total degree of PVDF crystallinity.

In contrast, the melt-quenched PVDF powder showed a total crystallinity of about 25%, remarkably similar to the original 26% of the as-received powder. However, melt-quenched PVDF also showed a significant increase in electroactive (β+γ) polymorph content, this fraction rising from 17% (as-received) to a massive 29% (*vide* Tables 3 and 4). This indicates that while the total crystallinity remained low, the melt-quenching process kinetically favored the formation of polar phases over the nonpolar α phase due to rapid solidification.

##### 3.5.3.2 Crystallinity Trends and PGMA Miscibility

In NIPS-blend membranes, PVDF crystallinity initially decreased with rising PGMA content (from 31% for 0PG to 24% for 30PG). This trend is attributed primarily to PGMA acting as a noncrystallizable diluent, which physically and kinetically impedes PVDF chain organization and crystal growth, overriding the effect of decreasing PVDF–PGMA attraction (increasing χ_12_) in this region (*vide* § 3.5.6). Crucially, λ_c_ subsequently increased sharply, reaching a notably high value (comparable to 0PG) at 50–60 vol % PGMA. This rise correlates strongly with the onset of macroscopic phase separation, allowing PVDF chains to aggregate and crystallize efficiently within their segregated domains. Finally, λ_c_ tended downward again, at very high PGMA concentrations, consistent with PVDF’s extreme kinetic confinement and dilution of the crystallizable PVDF component.

The crystallinity trend for the melt-quenched PVDF/PGMA blends generally mirrored that of the NIPS-formed samples (Figure 7), but with distinct quantitative differences. Crystallinity initially decreased with increasing PGMA concentrations (from 24% in 0PG to 20% in 30PG), then started to recover and peaked at about 30% in the 60PG blend, before decreasing again.

##### 3.5.3.3 Resolving the Kinetic Differences in Total Crystallinity

The NIPS-formed samples exhibited higher overall crystallinity compared to the melt-quenched versions (*cf*. Figure 7). This difference is rooted in the distinct kinetic environments of solidification.

The melt-quenching process, conducted at the maximum instrument cooling rate of 150 °C/min, represents an extreme kinetic suppression technique. While this rapid thermal gradient is sufficient to promote the formation of polar polymorphs (β, γ), it fundamentally restricts the time available for chain organization, resulting in a lower overall crystallinity (e.g., 25% for 0PG).

In contrast, the NIPS process allows for a relatively more prolonged solidification pathway. As the nonsolvent induces demixing, the PVDF polymer chains transition through a plasticized, solvent-swollen state. This diffusion-controlled phase separation provides chains with enhanced mobility and a greater duration of time for organization within the nascent polymer-rich phase, leading to a higher total degree of crystallinity (e.g., 31% for 0PG). This pathway allows the system to achieve a higher degree of total crystallization, even though the phase inversion front involves a rapid, localized change in composition that creates high shear/stress, which is essential for the strong promotion of the electroactive β-phase. The two mechanisms—rapid chain collapse for polymorphism, and prolonged plasticization for the overall extent of crystallization—are, therefore, complementary effects of the NIPS environment.

##### 3.5.3.4 Conclusion on Miscibility and Processing History

In summary, the crystallinity data from both NIPS-formed and melt-quenched blends strongly corroborate the multistage miscibility behavior inferred from other techniques. The initial decrease in crystallinity highlights the inhibitory effect of PGMA in a partially miscible state, while the subsequent increase in crystallinity for mid-range PGMA concentrations (peaking at 50% to 60% PGMA) provides clear evidence of phase separation, enabling more effective PVDF crystallization within segregated domains. Crucially, the differences in specific crystallinity values and transition points between the NIPS-formed and melt-quenched samples further underscore the significant role of processing history in dictating the final morphology and crystalline properties of these complex blends.

#### 3.5.4 Miscibility-Driven Kinetic Transitions in PVDF Dynamic Crystallization

Dynamic crystallization studies, conducted via a DSC cooling scan at 10 °C/min (Figure 6b, Table 4), revealed that the crystallization temperature (*T*_c_) of PVDF is profoundly sensitive to PGMA composition and blend miscibility. Initially, the neat PVDF membrane (0PG) exhibited an enhanced *T*_c_ of 134.6 °C—significantly higher than the commercial powder’s 131.2 °C—suggesting that the NIPS process imparted a structural history (e.g., residual β-phase nuclei) that boosted subsequent melt crystallization efficiency.

However, the *T*_c_ trend across the blends displayed a complex, multistage behavior that directly correlates with the transition from partial miscibility to macroscopic phase separation (Inset of Figure 6b). In the low PGMA region (0PG to 30PG), *T*_c_ consistently decreased due to kinetic hindrance. Here, PGMA acts as a noncrystallizable diluent within the miscible amorphous phase, impeding PVDF chain mobility and notably raising the overall *T*_g_ of the crystallizing melt, which necessitates greater undercooling. Beyond 30 vol % PGMA, the *T*_c_ exhibited a steeper drop, reaching a plateau around 123 °C for the 40PG and 50PG blends. This sudden depression and plateau signify the onset of liquid–liquid demixing (LLD) in the PVDF/PGMA blend. The sharp drop occurs because the macroscopic phase segregation required to form the PVDF-rich and PGMA-rich domains introduces a significant transient kinetic barrier to chain mobility. This structural reorganization successfully competes with and delays the subsequent solid–liquid demixing (crystallization) process during the dynamic cooling scan. Finally, at high PGMA loadings (>50 vol %), the *T*_c_ continued its sharp decline, dropping by a total of 28 °C relative to the neat PVDF membrane. This steep drop of the *T*_c_ in the final stage indicates extreme dilution and confinement, where the PVDF is kinetically trapped within the dominant PGMA matrix, severely restricting chain diffusion and the formation of crystalline structure, even after macroscopic phase separation has occurred.

#### 3.5.5 Crystalline Imperfection and Morphology as a Function of PGMA Content and Thermal History

The qualitative features and breadth of DSC melting endotherms (initial scans in Figure 5a and reheating scans in Figure 6a), quantitatively supported by Full Width at Half Maximum (fwhm) data (Figure 8), offer insights into PVDF crystal perfection and reorganization kinetics.^106^

**Figure 8:**
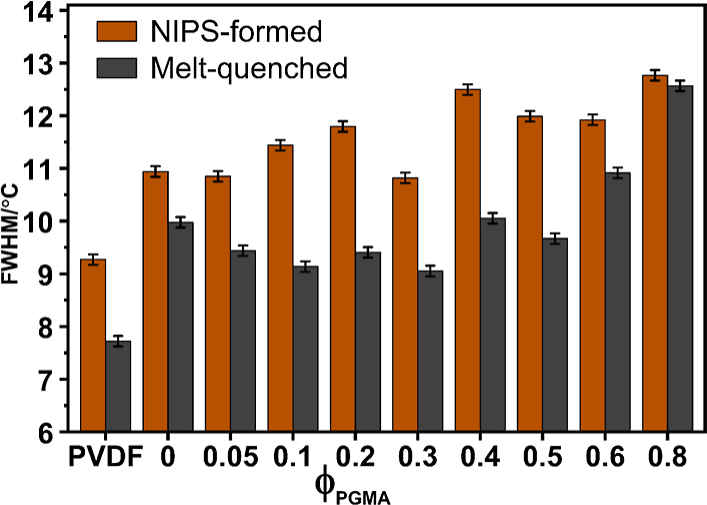
Full Width at Half Maximum (fwhm) values from DSC heating thermograms for NIPS-formed and melt-quenched samples of PVDF powder and PVDF/PGMA blends, presented as a function of PGMA volume fraction.

The as-received PVDF powder exhibited a melting endotherm with a low-temperature shoulder, signifying crystallite heterogeneity from its manufacturing process. However, melt-quenching the powder successfully erased this history, yielding a relatively sharp, single melting endotherm upon reheating (compare Figures 5a and 6a), which suggests a relatively uniform crystal population was formed by rapid cooling from the melt.

In contrast, the NIPS-formed neat PVDF membrane (0PG, Figure 5a) showed a broad single endotherm with significant low-temperature tailing. This is characteristic of a wide distribution of lamellar thicknesses and highly imperfect, kinetically trapped crystallites. This phenomenon is a direct consequence of the rapid, nonequilibrium thermodynamic quench inherent to the NIPS process, where the fast phase separation kinetics restrict molecular rearrangement, leading to high nucleation density but restricted crystal growth and perfection.^107^

To assess the impact of melt-state miscibility on the blends, NIPS-blends were subjected to melt-quenching and remelted in the DSC (Figure 6a). All melt-quenched samples, including neat PVDF, consistently displayed low-temperature tailing, confirming that rapid cooling from the melt universally produces a continuous distribution of smaller, less perfect crystals.

Specifically, in low PGMA-containing melt-quenched blends (up to 30 vol %), the endotherms showed a distinct low-temperature shoulder. This feature signifies a melting–recrystallization–remelting phenomenon, where less stable, small crystals reorganize during the scan.^78^ Importantly, beyond 30 vol % PGMA, this distinct shoulder became undetectable. Instead, the endotherms broadened significantly and showed more pronounced tailing, qualitatively resembling the NIPS-formed counterparts at high PGMA volume fractions.

Despite this broadening at high PGMA, the melt-quenched samples generally remained sharper than their NIPS-blend counterparts, as confirmed by the quantitative fwhm data (*cf*. Figure 8). This feature indicates that while the kinetic confinement caused by increasing PGMA loading leads to broader melting ranges, the initial melt-quenched state still resulted in comparatively more ordered PVDF crystals than the extreme nonequilibrium conditions of the NIPS process.

#### 3.5.6 Flory–Huggins Interaction Parameter (χ_12_) of PVDF/PGMA Blends

As mentioned in the context of the Gibbs–Thomson equation (*vide* § 3.5.1), polymer crystals formed under ordinary conditions have finite size and imperfections. Consequently, they melt at temperatures considerably lower than their true equilibrium melting temperature (*T*_m_^0^), which corresponds to the melting of a perfect, infinitely large crystal. The Hoffman–Weeks method,^85^ one of the most convenient of several extrapolation methods, was applied to determine the equilibrium melting points (*T*_m_^0^) of PVDF/PGMA blends. Figure 9 shows representative Hoffman–Weeks plots, and the corresponding *T*_m_^0^ values have been presented in Table 5.

**Figure 9:**
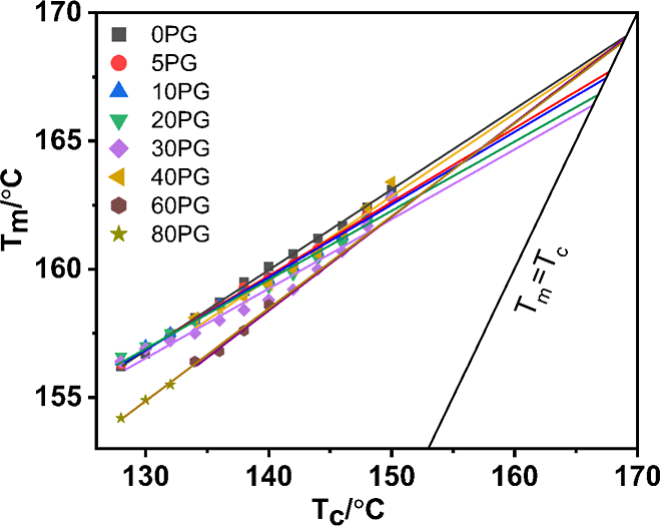
Representative Hoffman–Weeks plots of isothermally crystallized PVDF and PVDF/PGMA blends.

**Table 5:** Equilibrium Melting Points of PVDF Derived from Hoffman–Weeks Plots, and Interaction Parameters Calculated by the Nishi–Wang Method for PVDF/PGMA Blends (*r* ^2^ is the Coefficient of Determination in Linear Regression)

sample	*T*_m_^0^ (±0.2)/°C	*r* ^2^	ϕ_1_	χ_12_ (±0.01)
0PG	169.1	0.99	—	—
5PG	167.7	0.99	0.04946	–8.44
10PG	167.4	0.99	0.09809	–2.52
20PG	166.8	0.98	0.1969	–0.88
30PG	166.4	0.96	0.2961	–0.46
40PG	168.9	0.98	0.3956	–0.02
50PG	168.9	0.99	0.4956	–0.01
60PG	168.9	0.97	0.6024	–0.01
80PG	168.8	0.99	0.7968	–0.01

The *T*_m_^0^ is an essential thermodynamic parameter for studying the kinetics of polymer crystallization and for the determination of the Flory–Huggins interaction parameter (χ_12_) in polymer blends. In the present context, the determination of the χ_12_ parameter is crucial because it provides a quantitative measure of the miscibility or compatibility between two polymers. A negative χ_12_ value indicates favorable interaction and miscibility, whereas a positive χ_12_ value indicates unfavorable interaction and immiscibility. A χ_12_ value close to zero suggests borderline miscibility. In this study, the χ_12_ parameter was determined by the Nishi–Wang method.^108^ It relates the χ_12_ parameter with the depression of the equilibrium melting point of a semicrystalline polymer in a blend with an amorphous polymer as a function of the blend composition, by the following simplified equation^33,51^

10 $$\frac{1}{T_{mb}^{0}}-\frac{1}{T_{m}^{0}}=-\frac{RV_{2}}{\Delta H_{u_{2}}^{0}V_{1}}\chi_{12}\phi_{1}^{2}$$

here, 1 refers to the amorphous polymer component, PGMA, and 2 refers to the semicrystalline component, PVDF, in the present case; *T*_m_^0^ and *T*_mb_^0^ are the equilibrium melting points of PVDF in the neat state and in blends, respectively; *R* is the universal gas constant; *V*_*i*_ represents the molar volume per repeating unit of the component polymers, $\Delta H_{u_{2}}^{0}$ is the enthalpy of fusion per repeating unit of a perfect crystal of PVDF, and ϕ_1_ is the volume fraction of PGMA in the blend. The calculated χ_12_ values have also been displayed in Table 5.

Figure 10a displays the plot of the left-hand side of the Nishi–Wang equation (eq 10) against ϕ_1_^2^. This plot yielded a straight line with a small intercept on the *y*-axis for data points up to 30 vol % PGMA. The presence of the small intercept suggests a potential composition dependency of the χ_12_ parameter. The χ_12_ value calculated from the least-squares slope of this linear region was −0.209 ± 0.013, which is comparable to negative values reported for other miscible PVDF blends with polymers like poly­(1,2-butylene adipate) (−0.19),^52^ poly­(pivalolactone) (−0.13)^109^ and poly­(ethylene terephthalate) (−0.14).^33^

**Figure 10:**
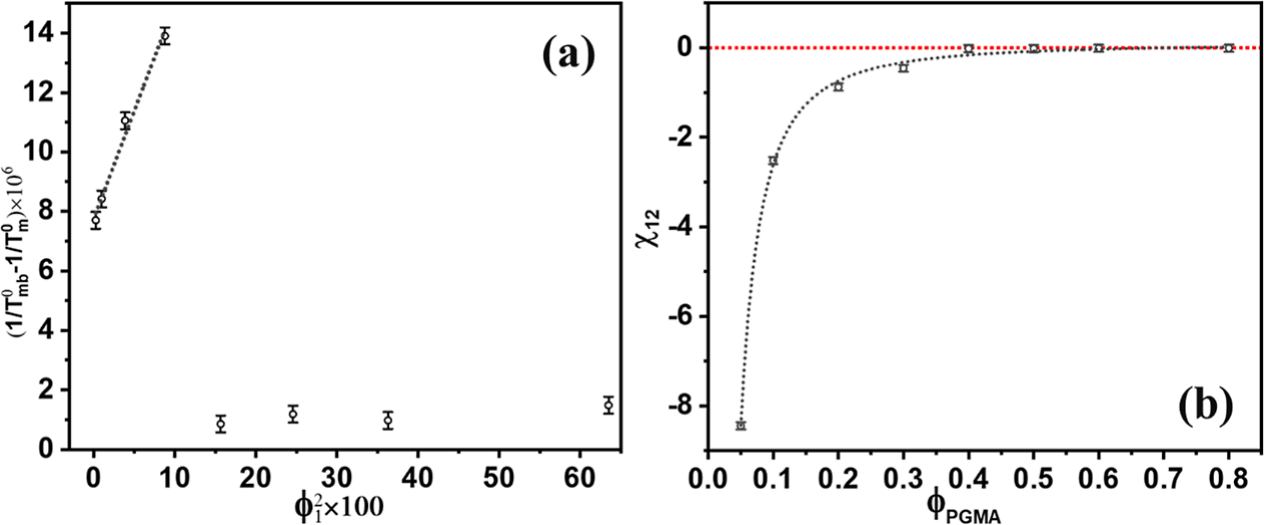
(a) Plot of the Nishi–Wang equation, and (b) χ_12_ as a function of the volume fraction of PGMA for PVDF/PGMA blends.

The χ_12_ values calculated for individual blend compositions according to the Nishi–Wang equation have been collected in Table 5, and their composition dependency is plotted in Figure 10b. The χ_12_ values were significantly negative for blends with less than 40 vol % PGMA concentration. Beyond this threshold, the χ_12_ value became close to zero. This transition from negative χ_12_ (indicating favorable interaction and miscibility) to near-zero χ_12_ (suggesting limited or no favorable interaction) confirms that the PVDF/PGMA is a partially miscible blend system with a miscibility window extending near to 40 vol % PGMA dilution, almost coinciding with the 37 vol % predicted by the Schneier equation. This conclusion is consistent with the phase behavior inferred from the XRD and FTIR analysis of the blends. Furthermore, the agreement of these findings validates the applicability of the Schneier equation for predicting the miscibility of this system.

### 3.6 Morphological Transition across the PVDF/PGMA Miscibility Window

Figure 11 presents FESEM images of the lateral surface cross-section of the PVDF/PGMA blend membranes, all confirming a highly porous structure. The pristine PVDF membrane (0PG) and low PGMA content blends (up to 30 vol %) exhibit a uniform, interconnected cellular structure characterized by a honeycomb-like pore network with smooth pore walls and regular pore distribution. This morphological uniformity strongly correlates with the miscible or partially miscible regime confirmed by the thermodynamic studies. The favorable specific interactions between the PVDF and PGMA segments in the dope solution promote a more controlled and homogeneous demixing, leading to the formation of a fine, regular, and well-integrated porous architecture during the NIPS process.

**Figure 11:**
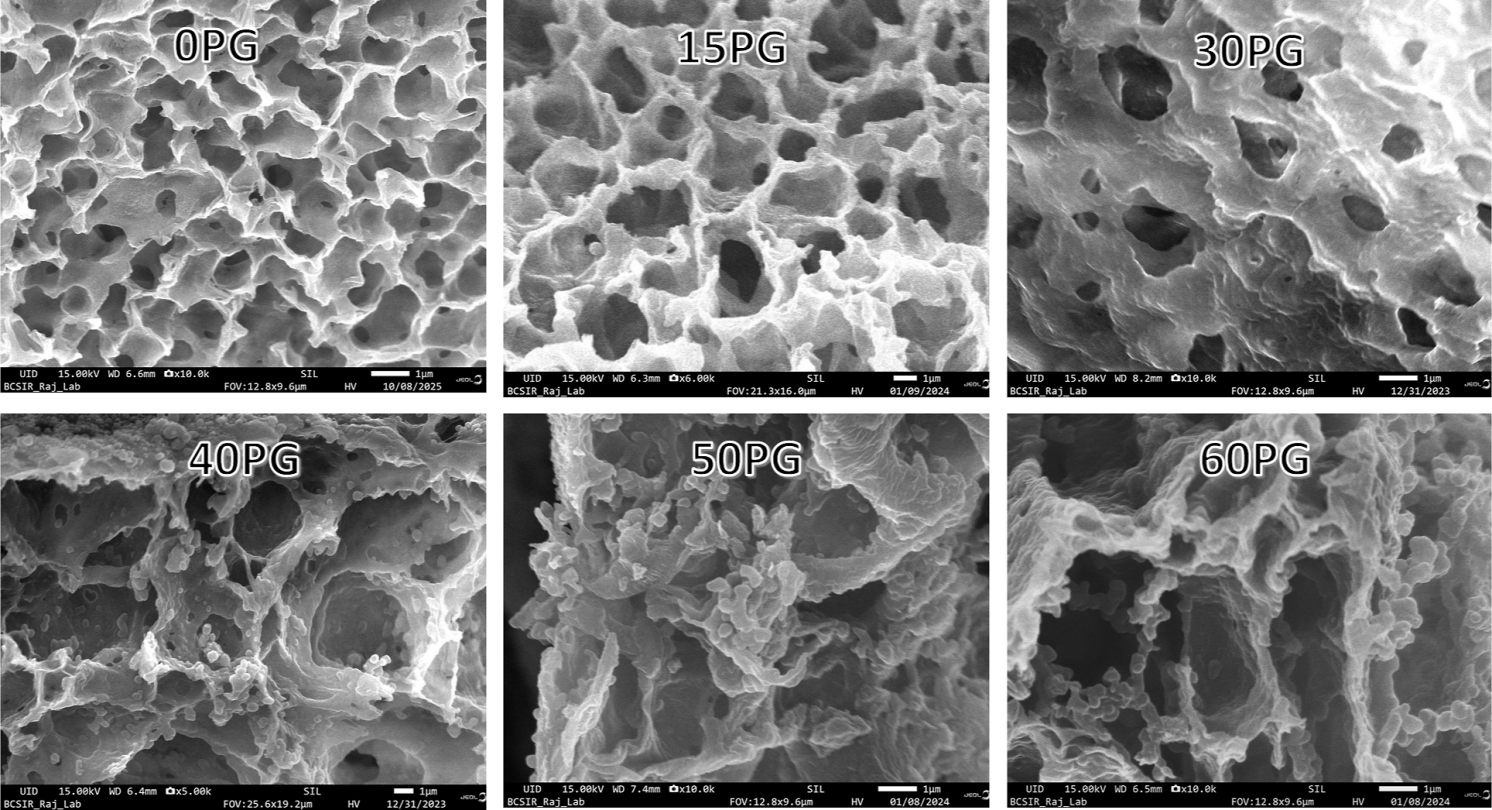
Representative FESEM images of the fractured surfaces of PVDF/PGMA membranes of different compositions as shown in the images. [scale bar = 1 μm].

In sharp contrast, membranes with higher PGMA content (beyond 30 vol %) exhibit a markedly heterogeneous morphology, characterized by an uneven distribution of macrovoids, large hollow channels and irregular pockets. Furthermore, the smooth pore walls transform into a pronounced granular texture. This contrasting morphology is a direct consequence of the onset of macroscopic phase separation beyond the critical PGMA concentration predicted by the thermodynamic miscibility studies. At these higher loadings, the increased segregation of PVDF and PGMA promotes less uniform pore nucleation and growth during the NIPS process, as the two phase-separated domains compete to form the final solid structure.

### 3.7 Porosity Enhancement: The Interplay of Crystallinity and Macroscopic Phase Separation

The gravimetrically determined porosity exhibited a sharp and linear increase from 76% (neat PVDF, 0PG) to approximately 87% at 50 vol % PGMA (Figure 12), a trend closely paralleled by the increase in average pore size. This consistent enhancement confirms that PGMA acts as a potent pore-former throughout the measured compositions. (Data collection for porosity was limited to 50 vol % PGMA, as higher concentrations resulted in mechanically brittle, nonfreestanding membranes.)

**Figure 12:**
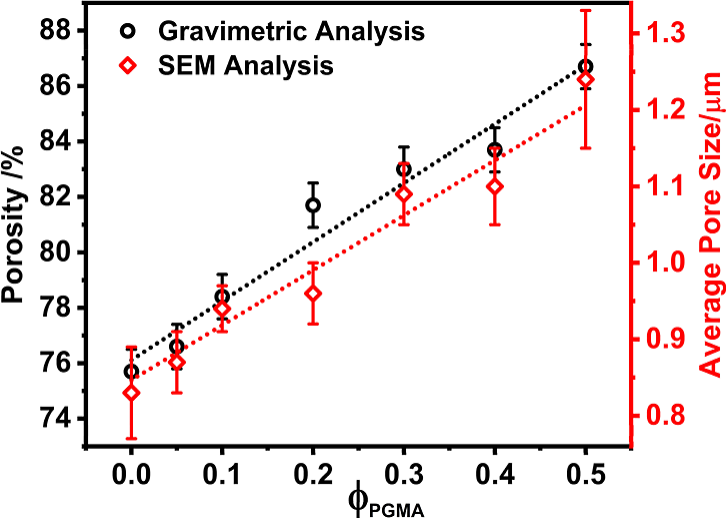
Membrane porosity (determined by gravimetry) and average pore diameter (determined from SEM image analysis via ImageJ software) as a function of the PGMA volume fraction (ϕ_PGMA_) in the PVDF/PGMA blends. Note: Beyond the 50 vol % PGMA composition, the membranes’ diminished mechanical integrity precluded effective gravimetric analysis.

The observed porosity increase is driven by a complex interplay of PVDF chain packing (crystallinity) and structural coarsening (macroscopic phase separation).

In the partially miscible regime (0PG–30PG), the porosity increase is primarily driven by reduced PVDF crystallinity. PGMA, acting as a noncrystallizable blending partner, interferes with the orderly alignment and close packing of PVDF chains, leading to a 23% reduction in PVDF crystallinity (from 31% at 0PG to a minimum of 24% at 30PG). This restriction of chain motility and close packing increases the internal void volume, thereby promoting overall porosity.

A critical and contradictory transition occurred at higher PGMA content: the PVDF crystallinity surprisingly increased, reaching as high as 33% at 50PG (*cf*. Table 3). Despite this localized increase in crystallinity, the overall membrane porosity continued its linear increase to 87%. This paradox is explained by the dominance of macroscopic phase separation (LLD). While the PVDF within its newly segregated domains can crystallize more efficiently (leading to a higher localized λ_c_), the overall NIPS process, now strongly driven by LLD, creates significantly coarser structural features. These large, irregular pockets and hollow channels (FESEM, Figure 11) become the primary determinant for the sharp increase in overall membrane porosity. Furthermore, the significant density difference between the polymers (ρ_PVDF_ ≈ 1.78 g/cm^3^ vs ρ_PGMA_ ≈ 1.09 g/cm^3^)^110,111^ means that replacing the denser PVDF with the less dense PGMA promotes a greater void fraction upon solvent removal from the PGMA-rich regions, overriding the localized effect of increased crystallinity.

### 3.8 Membrane Wetting Analysis: PGMA-Induced Hydrophilicity

The water contact angle (CA) measurements, rigorously evaluated across the full composition range (Figure 13), display a pronounced, multistage increase in membrane hydrophilicity with increasing PGMA content, presented in Table 6. This effect is driven by the presence of PGMA’s carbonyl (C=O) groups, which readily interact with water via hydrogen bonding.

**Figure 13:**
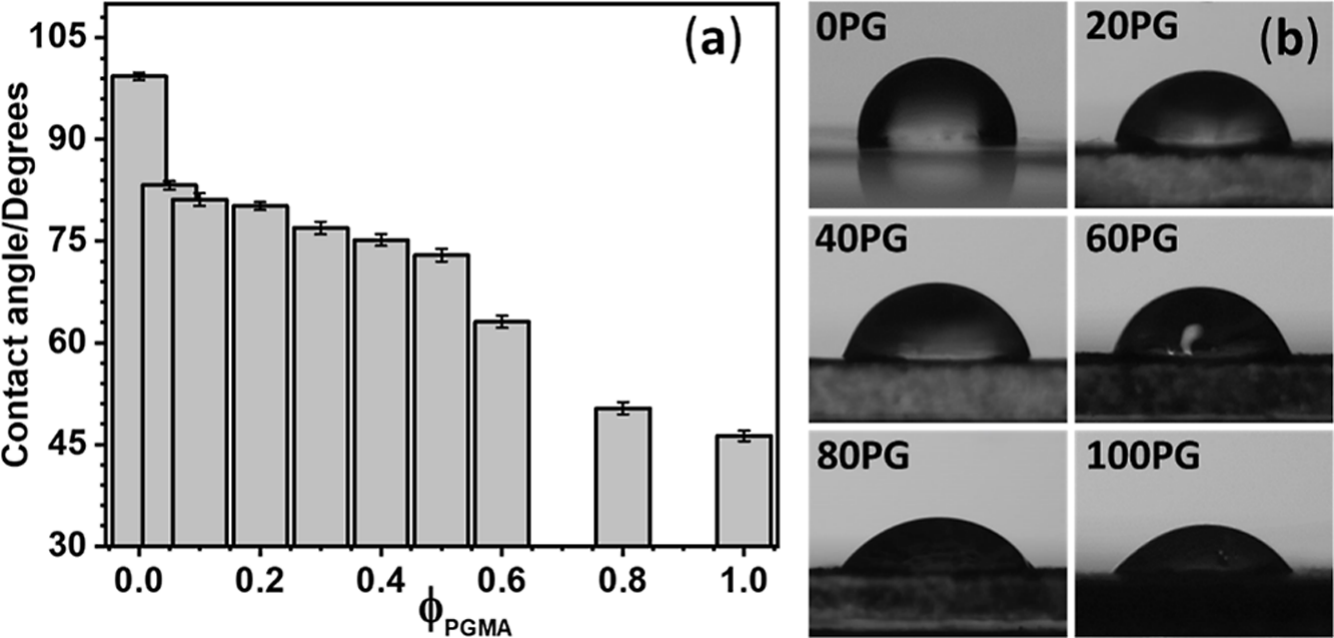
(a) Average contact angle vs volume fraction of PGMA in PVDF/PGMA blends, and (b) Representative photographs of water droplets from which the contact angles were estimated.

**Table 6:** Distinct Stages of Wetting Behavior Reflecting the Underlying Thermodynamic and Morphological Changes

composition	CA change (trend)	underlying mechanism
0PG-5PG	sharp drop (99.3° to 83.2°)	Rapid Surface Enrichment: A minute amount of the inherently hydrophilic PGMA efficiently segregates to the membrane surface during NIPS, causing an immediate and significant change in surface energy
5PG-50PG	slower linear decrease (83.2° to 72.9°)	Phase Competition: As the blends transition toward macroscopic phase separation, the rate of hydrophilicity improvement slows. The increasingly coarse and segregated morphology (observed via FESEM, Figure 11) may sequester some PGMA in bulk domains, leading to less efficient exposure of hydrophilic groups at the immediate surface
>50PG	steep decrease (to 46.2° for neat PGMA)	Bulk Dominance: PGMA becomes the dominant component. Despite significant phase separation, the sheer abundance of hydrophilic PGMA overwhelms the surface, leading to a dramatic increase in wettability, approaching the neat PGMA value

This successful and controllable enhancement of surface hydrophilicity is critical. PVDF’s inherent hydrophobicity often leads to membrane fouling in water treatment and biomedical applications. Tailoring the surface CA via PGMA incorporation provides a powerful strategy to mitigate fouling, enhance water flux, and improve the operational stability of these membranes for diverse separation and biomaterial requirements.^112^

## 4 Conclusion

This study presents a systematic investigation into the miscibility and morphological evolution of poly­(vinylidene fluoride) (PVDF)/poly­(glycidyl methacrylate) (PGMA) blends fabricated into membranes via the NIPS process. Our comprehensive analysis, spanning spectroscopic, thermal, and morphological techniques, firmly established that the system exhibits partial miscibility in both as-prepared and melt states, with a critical miscibility boundary, corroborated by theoretical predictions, positioned at approximately 37 vol % PGMA.

In the miscible regime (up to ∼30 vol % PGMA), strong interpolymer interactions were evidenced by significant melting point depression and highly negative Flory–Huggins interaction parameters. Crucially, while these interactions slightly suppressed the overall PVDF crystallinity (declining from 31% at 0PG to 24% at 30PG), they provided a powerful driving force for the formation of the electroactive β-phase, with the 10PG blend achieving an impressive 70% β-phase content. Beyond the miscibility threshold, macroscopic phase separation became dominant, consistent with the recovery in melting temperature and crystallinity values. The nonmonotonic trends in crystallinity (DSC) and lamellar thickness (XRD) served to structurally validate the transition from a miscible to a phase-separated regime.

Furthermore, PGMA incorporation effectively modulated the membrane structure, yielding significant enhancements in porosity and surface hydrophilicity, alongside promoting uniform membrane morphology at lower concentrations. The demonstrated ability to tune the PVDF microstructure and surface properties simply by blend composition and processing route highlights the strong potential of these novel membranes for advanced electroactive and functional biomedical applications.

While this work provides essential, fundamental insights into the structure–property relationships of PVDF/PGMA blends, future research may focus on elucidating the precise nucleation mechanisms of PVDF polymorphs within these complex blend environments. Additionally, evaluating the long-term stability and commercial scalability of the membranes will be crucial for their successful translation to real-world contexts.

## Supplementary Material



## Notes

GPC chromatogram and molecular weight distribution of PGMA (Figure S1; Table S1); intrinsic viscosity and viscosity-average molecular weight determination of PGMA (Figure S2); XRD-derived interplanar distance and lamellar thickness of PVDF crystals in PVDF/PGMA blend membranes (Table S2); detailed calculation procedure for β-phase fraction, F­(β), in PVDF/PGMA blends; FESEM-based membrane thickness and pore size statistics (Table S3), pore size distribution curves (Figure S3), and digital photographs of membranes (Figure S4) (PDF)
