# Supplementary material for: PVDF/PGMA Blend Membranes: NIPS-Driven Microstructure, Thermodynamic Miscibility, and Enhanced Wettability
Source: ACS Omega. 2026 Jan 21;11(4):5450–67. doi: 10.1021/acsomega.5c08866 (PMC12878725; doi:10.1021/acsomega.5c08866)
Supplement: Supplementary file 1 [file ao5c08866_si_001.pdf]

Supporting Information (SI)

PVDF/PGMA Blend Membranes: NIPS-Driven Microstructure, Thermodynamic Miscibility, and Enhanced Wettability

Md. Azizul Hakim [a], Md. Mahadi Hasan [a], Md. Al-Mamun [b,c], Md. Shamim Hossan [a], A.A.S. Mostofa Zahid [a], and M. Habibur Rahman [a,*]

[a] *Department of Chemistry, University of Rajshahi, Rajshahi 6205, Bangladesh*

[b] *Materials Science Division, Atomic Energy Center, Bangladesh Atomic Energy Commission, Dhaka 1207, Bangladesh*

[c] *Department of Nutrition and Food Engineering, Daffodil International University, Birulia, Dhaka 1216, Bangladesh*

***Corresponding Author**

Email: mhr@ru.ac.bd (MHR)

S1. GPC Chromatogram and Molecular Weight Distribution of PGMA

This section presents the GPC chromatogram of the synthesized PGMA along with the corresponding molecular weight distribution parameters, as discussed in the main manuscript *(Section 2.2)*.

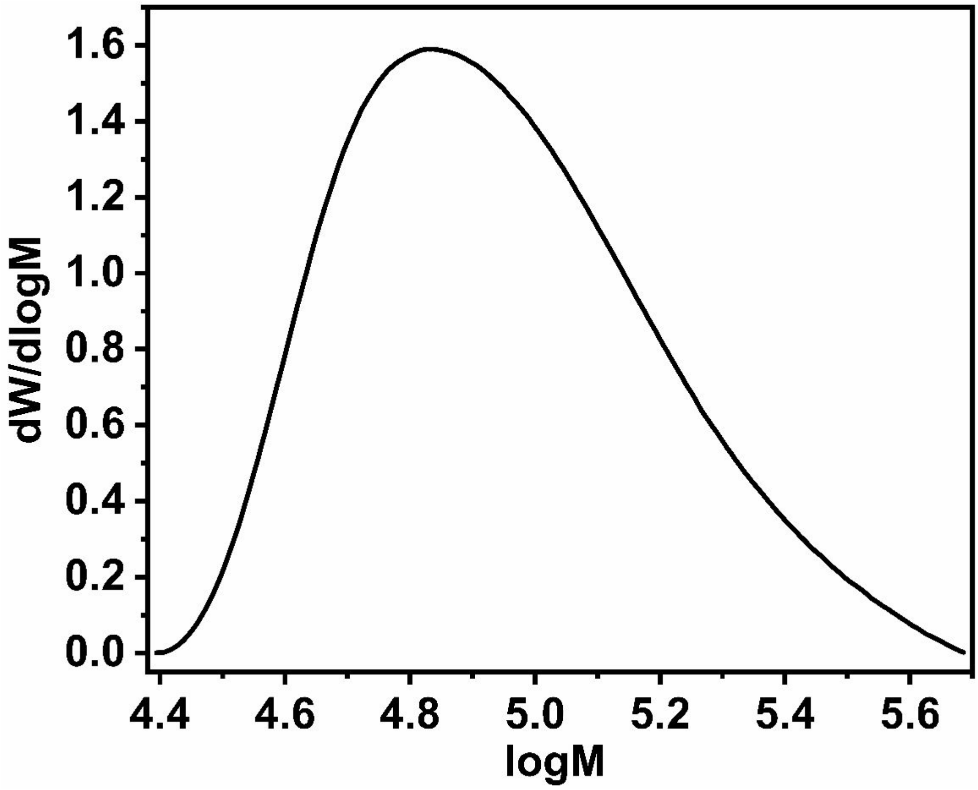

Figure S1. Gel Permeation Chromatography (GPC) trace of Poly(glycidyl methacrylate) (PGMA), showing the molecular weight distribution (dW/dlogM vs. logM). The broad, non-symmetrical distribution is entirely consistent with PGMA polymerization via conventional free radical kinetics using the AIBN as initiator at 60 °C. The broadness indicates an uncontrolled process governed by random radical termination, yielding a polymer with a high PDI. The high-molecular-weight tailing suggests that chain coupling is a significant termination pathway, or that chain transfer to polymer is occurring.

Table S1. Summary of Gel Permeation Chromatography (GPC) Data for Poly(glycidyl methacrylate) (PGMA): Molecular Weights and Polydispersity.

Sample	Elution Time (min)	$\overline{M}_n$ (kDa)	$\overline{M}_p$ (kDa)	$\overline{M}_w$ (kDa)	$\overline{M}_z$ (kDa)	$\overline{M}_v$ (kDa)	PDI ($\overline{M}_w/\overline{M}_n$)
PGMA	22.74	80.2	75.1	108.8	151.6	103.7	1.36

S2. Determination of Intrinsic Viscosity and Molecular Weight of PGMA

This section provides the detailed experimental procedure, theoretical background, and calculation steps used for determining the intrinsic viscosity and viscosity-average molecular weight (M_v) of PGMA, as referenced in the main manuscript *(Section 2.2)*. For Intrinsic Viscosity Measurements, PGMA was dissolved in THF by stirring overnight at 70 °C in an oil bath and subsequently cooled down to 30 °C, where the stirring was continued for 24 hours at 300 rpm. The intrinsic viscosity of the polymer was determined from the viscosity data of a series of dilute THF solutions of the polymer measured by an Ostwald viscometer at 30 °C using Equation 1.[1]

$$[\eta] = \lim_{c \to 0} \frac{\eta_{sp}}{c} \qquad (1)$$

The plot of reduced viscosity $\left(\frac{\eta_{sp}}{c}\right)$ as a function of concentration was linear with the coefficient of determination of 0.98 and gave an intercept of 0.13256 ± 0.015 dL/g when extrapolated to zero concentration, which by definition, is the intrinsic viscosity, [η]. The viscosity average molecular weight ($\overline{M}_v$), which closely aligns with the weight average molecular weight ($\overline{M}_w$) of the polymer,[1] was obtained using the Mark-Houwink-Sakurada (MHS) relation (Equation 2).

$$[\eta] = K\overline{M}_v^{\alpha} \qquad (2)$$

Here, Literature values of the Mark-Houwink parameters 'K' and 'α' at 30 °C for the PGMA-THF system, 2.78×10^{-4} dL/g and 0.537, respectively, were used for the calculation,[2] yielding $\overline{M}_v \cong \overline{M}_w$ of 97.2 ± 11.0 kDa.

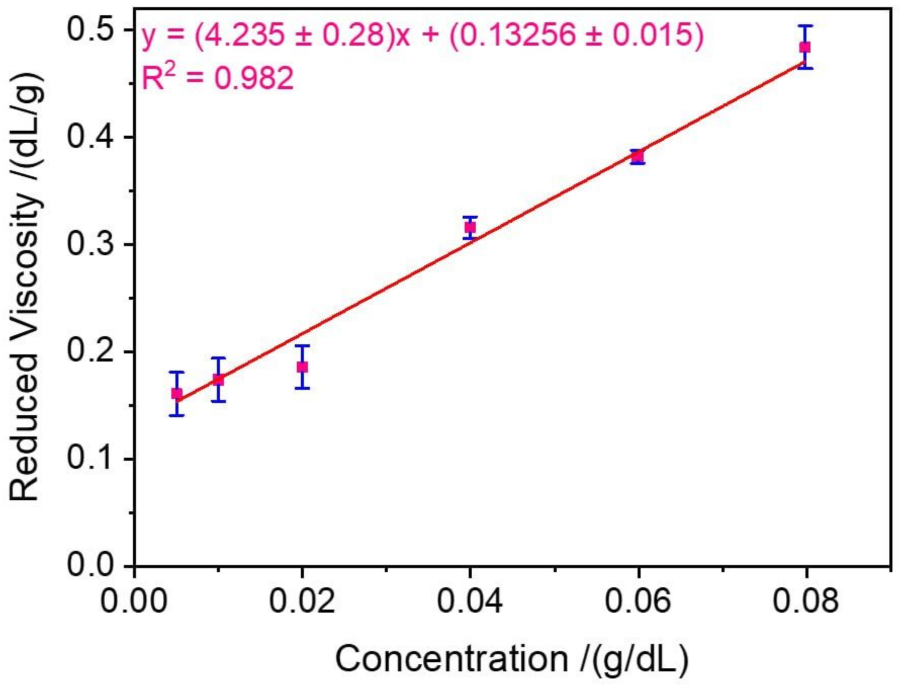

Figure S2. Plot of Reduced Viscosity vs. Concentration of PGMA Solutions in THF at 30 °C with Linear Fit.

S3. Interplanar Distance and Lamellar Thickness of PVDF Crystals in the Blend Membranes: XRD analysis

To complement the structural analysis presented in the main manuscript *(Section 3.2)*, the detailed XRD data used to calculate the interplanar distance and lamellar thickness of PVDF crystals in the blend membranes are provided here.

The interplanar distance or *d*-spacing was calculated by the Bragg Equation (3).[3]

$$d_{h\,k\,l} = \frac{\lambda}{2\ sin\theta} \quad (3)$$

Here, $\boldsymbol{\lambda}$ = 0.1542 nm, and θ is the Bragg angle.

The lamellar thickness *L* was calculated using the Scherrer Equation (4).[3]

$$L = \frac{k\lambda}{\beta\ cos\theta} \quad (4)$$

Here, k = 1.0, and β represents the full width at half maximum (FWHM) of the Bragg reflection corresponding to the specific crystallographic plane. [All the XRD data were analyzed by using OriginPro 2018 64-bit software]

Table S2. Interplanar distance (*d* value) and lamellar thickness (*L* value) of PVDF crystals in the powder form (α-crystals) and in the blend membranes (β-crystals) as estimated from the diffraction maxima in the region of 2θ = 20.3° (110); δ represents the standard error in the data.

Sample	$(2\theta \pm \delta 2\theta)$/°	(d ± δ*d*)/nm	(*L* ± δ*L*)/nm
PVDF	19.92 ± 0.08	0.45 ± 0.01	17.65 ± 0.11
0PG	20.34 ± 0.06	0.44 ± 0.01	7.73 ± 0.14
5PG	20.24 ± 0.04	0.44 ± 0.01	5.88 ± 0.47
10PG	20.36 ± 0.04	0.44 ± 0.01	6.67 ± 0.35
20PG	20.37 ± 0.08	0.44 ± 0.01	5.49 ± 0.27
30PG	20.40 ± 0.10	0.44 ± 0.02	4.76 ± 0.58
40PG	20.20 ± 0.06	0.44 ± 0.01	7.21 ± 0.34
50PG	20.18 ± 0.08	0.44 ± 0.01	6.69 ± 0.55
60PG	20.10 ± 0.06	0.44 ± 0.01	7.63 ± 0.71
80PG	20.12 ± 0.06	0.44 ± 0.01	5.71 ± 0.52

The results of this analysis, along with those for the PVDF powder, are summarized in Table S2. Notably, the interplanar distance remained consistent across the PVDF powder and all the prepared membranes, implying that the unit cell dimensions and the arrangement of PVDF molecules within the crystal lattice planes were preserved.

However, a significant reduction in the lamellar thickness (*L*) was observed in the membranes compared to the powder. Specifically, the *L* value of the neat PVDF membrane (0PG) remarkably decreased to 7.7 nm from 17.6 nm of the commercial PVDF powder. This substantial reduction is presumed to be a characteristic consequence of the non-solvent induced phase separation (NIPS) process. The rapid solvent/non-solvent exchange inherent in NIPS leads to fast polymer precipitation, kinetically hindering the formation of thicker, more stable lamellae. This rapid process also restricts PVDF chain mobility and promotes a high density of nucleation sites, resulting in numerous smaller crystallites with thinner lamellae. The high polymer concentration in the dope solution could also restrict the free diffusion of chains necessary for optimal crystal growth.

For the PVDF/PGMA blend membranes, the *L* values exhibited a distinct, non-monotonic three-stage trend, reflecting the evolving polymer interactions and phase behavior:

Low PGMA (0 – 30% v/v) Content Blends: In this range, the L value gradually decreased from 7.7 nm (0PG) to 4.8 nm (30PG) (Table S2). This consistent reduction suggests that the initial introduction of PGMA, within the partially miscible regime, increasingly hinders the crystallization of PVDF into thicker lamellae. This is likely due to growing interpolymer interactions between PVDF and PGMA chains and alterations in the phase separation kinetics during membrane formation, disrupting PVDF's ability to form well-ordered, thicker lamellae.

Mid PGMA (40 – 60% v/v) Content Blends: A significant change occurred in this compositional range, where the *L* value increased abruptly and plateaued at a level comparable to 0PG (around 7 nm) (Table S2). This recovery in lamellar thickness strongly indicates the onset of macroscopic phase separation. At these concentrations, PVDF-rich and PGMA-rich domains begin to form more distinctly, allowing PVDF chains to crystallize more effectively within their segregated, purer environments, forming thicker lamellae.

High PGMA (>60% v/v) Content Blends: In this third group, the *L* values tended to decrease again (Table S2). This subsequent reduction suggests that at very high PGMA loadings, despite ongoing phase separation, the extreme dilution of PVDF and the increasing dominance of the PGMA matrix significantly restrict the mobility of PVDF chains and disrupt its ability to form thicker lamellae, leading to overall thinner crystal domains.

The non-monotonic change in lamellar thickness (L) with increasing PGMA content in the blend membranes provides compelling evidence for the partial miscibility of the PVDF/PGMA system. This behavior conspicuously mirrors trends observed in DSC data and is consistent with a miscibility gap predicted by the Schneier equation at PGMA concentrations above approximately 37% (v/v).

S4. Calculating the β-phase fraction of PVDF (*F(β)*) in a PVDF/PGMA Blend

To support the FTIR analysis discussed in *Section 3.3.2* of the main manuscript, the detailed procedure for calculating the β-phase fraction, F(β), in the PVDF/PGMA blend membranes is provided here. A modified form of the Gregorio-Cestari Equation[4] for calculating the β-phase fraction, F(β), in a PVDF/PGMA blend has been used:

$$F(\beta) = \frac{A_{\beta,true}/K_\beta}{A_{\alpha,true}/K_\alpha + A_{\beta,true}/K_\beta} \quad (5)$$

Here, $A_{\alpha,true}$ is the true absorbance of the PVDF α-phase peak (at 764 cm^{-1}) and $A_{\beta,true}$ is the true absorbance of the PVDF β-phase peak (at 840 cm^{-1})

To find these "true" absorbances, we first calculate the absorbance contributed by PGMA at each position using a reference PGMA peak that does not overlap with any PVDF peaks. In this case, we use the C=O absorption of PGMA at 1722 cm^{-1} as the normalization peak (ν_{norm}).

Estimate PGMA contributions: Calculate the absorbance contributed by PGMA at 764 cm^{-1} ($A_{PGMA}(764)$) and 840 cm^{-1} ($A_{PGMA}(840)$) by scaling the neat PGMA absorbances at 753 cm^{-1} and 843 cm^{-1}, respectively.

$$A_{PGMA}(764) = A_{100PG}(753) \times \frac{A_{xPG}(1722)}{A_{100PG}(1722)} \quad (6)$$

$$A_{PGMA}(840) = A_{100PG}(843) \times \frac{A_{xPG}(1722)}{A_{100PG}(1722)} \quad (7)$$

Calculate true PVDF absorbances: Subtract the PGMA contribution from the total measured absorbance of the blend at each position.

$$A_{\alpha,true} = A_{total}(764) - A_{PGMA}(764) \quad (8)$$

$$A_{\beta,true} = A_{total}(840) - A_{PGMA}(840) \quad (9)$$

Finally, substitute the "true" absorbance values into *Equation 5*.

$$F(\beta) = \frac{A_{total}(840) - A_{PGMA}(840)/K_\beta}{A_{total}(764) - A_{PGMA}(764)/K_\alpha + A_{total}(840) - A_{PGMA}(840)/K_\beta} \quad (10)$$

This calculation eliminates the effect of membrane thickness. It achieves this by using ratios of absorbances from the same membrane, rather than absolute absorbance values. The final Modified Gregorio-Cestari Equation in its familiar form is:

$$F(\beta) = \frac{A_{total}(840) - A_{PGMA}(840)}{1.3\left(A_{total}(764) - A_{PGMA}(764)\right) + \left(A_{total}(840) - A_{PGMA}(840)\right)} \quad (11)$$

This equation (11) was used to determine the ***(β+γ)***-phase content of PVDF in NIPS-formed and melt-quenched PVDF/PGMA membranes *(Tables 3 and 4, main manuscript).*

S5. Morphology of NIPS-Formed Membranes

The average membrane thickness and pore diameter (pore size) were determined from FESEM micrographs analyzed by ImageJ software.

Table S3. Average thickness (*l*), pore size (*ε*), and pore size range (Δε) of the NIPS formed PVDF/PGMA membranes, determined by FESEM image analysis (ImageJ software).

Sample	Average thickness, ($l \pm \delta l$) /µm	Average pore size, ($\varepsilon \pm \delta\varepsilon$)/µm	Pore size range, ($\Delta\varepsilon$)/µm
0PG	32.80 ± 0.81 (n = 6)	0.83 ± 0.06 (n = 69)	0.24 – 3.2
5PG	23.39 ± 0.36 (n = 4)	0.87 ± 0.04 (n = 109)	0.20 – 2.0
10PG	45.48 ± 0.34 (n = 5)	0.94 ± 0.03 (n = 102)	0.30 – 1.7
20PG	45.18 ± 0.65 (n = 8)	0.96 ± 0.04 (n = 101)	0.30 – 2.1
30PG	25.78 ± 0.02 (n = 7)	1.09 ± 0.04 (n = 102)	0.26 – 3.2
40PG	34.68 ± 0.61 (n = 6)	1.10 ± 0.05 (n = 107)	0.36 – 3.4
50PG	30.62 ± 0.21 (n = 7)	1.24 ± 0.09 (n = 63)	0.32 – 3.4
60PG	46.81 ± 0.21 (n = 6)	1.27 ± 0.10 (n = 83)	0.35 – 2.6

n is the sample size

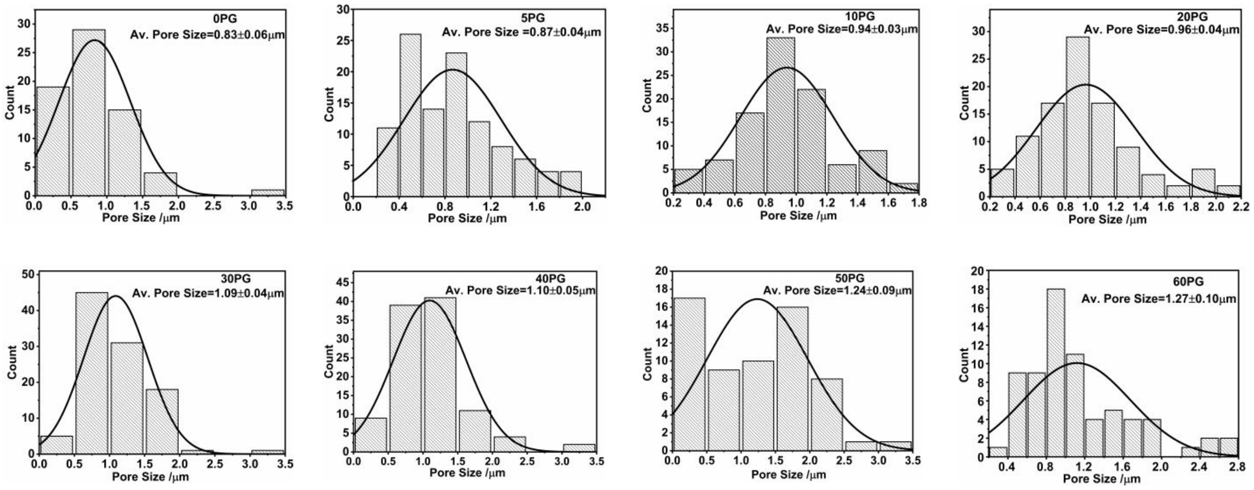

Figure S3. Pore Size Distribution (PSD) curves for PVDF/PGMA blend membranes, derived from analysis of FESEM images using ImageJ software.

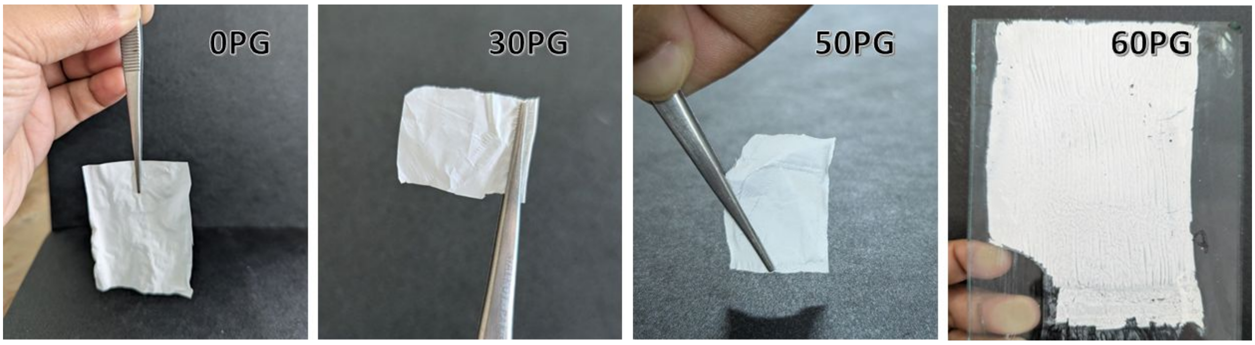

Figure S4. Representative digital photographs of neat PVDF and PVDF/PGMA blend membranes. The membranes containing up to 50% PGMA remained free-standing, as evident from the photographs. Membranes with PGMA content above 50% adhered to glass surfaces.

References

(1) Bottino, A.; Capannelli, G.; Munari, S.; Turturro, A. Solubility Parameters of poly(Vinylidene Fluoride). *J Polym Sci B Polym Phys* **1988**, *26* (4), 785–794. https://doi.org/10.1002/polb.1988.090260405.

(2) Hutchinson, R. A.; Beuermann, S.; Paquet, D. A.; McMinn, J. H.; Jackson, C. Determination of Free-Radical Propagation Rate Coefficients for Cycloalkyl and Functional Methacrylates by Pulsed-Laser Polymerization. *Macromolecules* **1998**, *31* (5), 1542–1547. https://doi.org/10.1021/ma971307u.

(3) Ma, W.; Zhang, J.; Wang, X.; Wang, S. Effect of PMMA on Crystallization Behavior and Hydrophilicity of Poly(Vinylidene Fluoride)/Poly(Methyl Methacrylate) Blend Prepared in Semi-Dilute Solutions. *Applied Surface Science* **2007**, *253* (20), 8377–8388. https://doi.org/10.1016/j.apsusc.2007.04.001.

(4) Gregorio, Jr., R.; Cestari, M. Effect of Crystallization Temperature on the Crystalline Phase Content and Morphology of Poly(Vinylidene Fluoride). *J Polym Sci B Polym Phys* **1994**, *32* (5), 859–870. https://doi.org/10.1002/polb.1994.090320509.
